# Ropivacaine Local Infiltration for Pain Control After Thyroidectomy: A Systematic Review and Meta‐Analysis

**DOI:** 10.1002/oto2.70124

**Published:** 2025-05-05

**Authors:** Ebraheem Albazee, Fahad Allafi, Abdulwahab Alsalem, Deemah AlShaya, Hayfaa Alhazami, Danah Alfalah

**Affiliations:** ^1^ Otorhinolaryngology–Head and Neck Surgery Kuwait Institute for Medical Specializations (KIMS) Kuwait City Kuwait; ^2^ Department of General Surgery, Al‐Jahra Hospital Ministry of Health Al Jahra Kuwait; ^3^ Department of General Surgery, Amiri Hospital Ministry of Health Kuwait City Kuwait; ^4^ Department of Emergency Medicine, Amiri Hospital Ministry of Health Kuwait City Kuwait

**Keywords:** analgesia, anesthesia, ropivacaine, surgery, thyroid, thyroidectomy

## Abstract

**Objective:**

To evaluate the analgesic role of ropivacaine local infiltration in patients undergoing thyroidectomy.

**Data Sources:**

PubMed, Google Scholar, CENTRAL, Scopus, and Web of Science.

**Review Methods:**

A systematic review and meta‐analysis synthesizing evidence from randomized controlled trials (RCTs). Our specific endpoints include pain severity, total opioid analgesia consumption, patient satisfaction, length of hospital stay, postanesthesia care unit (PACU) length of stay, surgery duration, and the incidence of postoperative nausea and vomiting (PONV). Using Stata, we pooled dichotomous outcomes and continuous outcomes using risk ratio (RR) and standardized mean difference (SMD) or mean difference (MD), respectively, with a 95% confidence interval (CI).

**Results:**

Eight RCTs and 633 patients were included. Ropivacaine significantly decreased pain after 1 to 2 hours postoperatively (SMD: −1.40, 95% CI [−2.30, −0.51]). However, there was no difference between both groups after 4 hours (*P* = .11), 6 to 8 hours (*P* = .05), 16 to 18 hours (*P* = .10), and 24 hours (*P* = .37). Also, ropivacaine significantly decreased analgesia consumption (SMD: −0.75, 95% CI [−1.30, −0.20]), with no effect on surgery duration (*P* = .59), length of hospital stays (*P* = .32), patient satisfaction score (*P* = .25), and PACU length of stay (*P* = .25). Finally, there was no difference between both groups regarding the incidence of PONV (RR: 1.01, 95% CI [0.70, 1.45]).

**Conclusion:**

Ropivacaine local infiltration after thyroidectomy significantly decreased pain for up to 1 to 2 hours and analgesia consumption compared to control, but with uncertain evidence. However, ropivacaine had no effect on pain from 4 to 24 hours, surgery duration, length of PACU stay, length of hospital stay, and patient satisfaction.

Thyroid surgery is a common procedure, with about 93,000 thyroidectomies performed annually in the United States.^1^ Patients may experience significant pain after thyroid surgery, especially in the first few hours.^2^, ^3^ Postthyroidectomy mean pain, measured on a 0‐ to 100‐mm visual analog scale (VAS), was reported at an average of 69,^4^ and another study reported values ranging from 55 to 78, contingent upon the intraoperative analgesia.^5^ Thyroid surgery and the associated need for analgesia are established risk factors for postoperative nausea and vomiting (PONV), a typical patient complaint that may elevate the risk of postoperative hemorrhage necessitating emergency intervention—a rare but potentially lethal complication.^4^, ^5^, ^6^, ^7^ Therefore, minimizing postoperative pain and opioid consumption is essential in the care of patients following thyroid surgery.^8^

Pain management strategies following thyroidectomy vary widely across the globe, influenced by regional practices, available resources, and clinical guidelines.^9^ These approaches differ in terms of timing (preoperative, intraoperative, and postoperative), type of analgesia (opioid‐based regimens, nonsteroidal anti‐inflammatory drugs [NSAIDs], local anesthetic infiltration, and nonpharmacologic methods such as warm or cold compresses), and whether a single or multimodal analgesic model is adopted.^9^ Postthyroidectomy analgesia is typically managed using a wide range of analgesics, including NSAIDs and opioid analgesics, which are the preferred choices.^10^, ^11^ Still, both options are associated with several adverse events, especially PONV.^12^ In general surgery, local wound infiltration has a long history of use to mitigate postoperative pain and decrease reliance on analgesics.^12^ In thyroidectomy, evidence shows that local wound infiltration is a feasible technique conducted by the surgeon, elongating the time until patients request rescue analgesia.^13^ Nevertheless, the optimal local anesthetic agent for this approach remains unclear. This highlights the global diversity in postthyroidectomy pain management protocols and underscores the need for evidence‐based guidance.

Ropivacaine, a long‐acting amide local anesthetic, offers a favorable safety profile, reduced cardiotoxicity compared to conventional bupivacaine, and selective sensory blockade that preserves motor function.^14^, ^15^ Administering ropivacaine to surgical sites offers focused pain management by blocking sodium channels and, thus, pain signals.^16^ Several randomized controlled trials (RCTs) have evaluated ropivacaine local infiltration to mitigate postthyroidectomy pain and decrease analgesia consumption.^7^, ^8^, ^12^, ^16^, ^17^, ^18^, ^19^

Nevertheless, they reported mixed results, and the summarized evidence has not been synthesized yet.^7^, ^8^, ^12^, ^16^, ^17^, ^18^, ^19^ Hence, we conducted this systematic review and meta‐analysis to synthesize the evidence from RCTs on the efficacy of ropivacaine in decreasing postthyroidectomy pain, analgesia consumption, and clinically related outcomes.

## Methodology

### Protocol Registration

Before peer review, this systematic review was registered with the International Prospective Register of Systematic Reviews (PROSPERO) via the CRD420251025841. This systematic review and meta‐analysis adhered to the Preferred Reporting Items for Systematic Reviews and Meta‐Analyses (PRISMA) statement^20^ and the *Cochrane Handbook for Systematic Reviews of Interventions*.^21^

### Data Sources and Search Strategy

In January 2025, an electronic search was conducted by E.A. and F.A. on the following databases: Web of Science (WOS), PubMed, Google Scholar, Scopus, and CENTRAL. The search strategy incorporated the following entry terms: “(thyroidectom* OR ‘total thyroidectomy’ OR ‘thyroid surgery’) AND (ropivacain* OR ‘ropivacaine hydrochloride’ OR ‘ropivacaine monohydrochloride’ OR naropeine OR naropin OR ‘LEA 103’ OR ‘LEA‐103’ OR ‘AL 381’ OR ‘1 Propyl 2′,6′ pipecoloxylidide’ OR ‘(S)‐Ropivacaine’ OR ‘84057‐95‐4’ OR ‘rocaine’ OR ‘local anaesthesia’ OR ‘local anesthesia’ OR ‘local analgesia’ OR ‘local anesthetic’ OR ‘local anaesthetic’)”. Our search was unconstrained, except for Scopus, where we limited the search scope to titles, abstracts, and keywords. Each database's entry terms and search results are demonstrated in Supplemental Table S1, available online. To ensure a complete review and avoid the exclusion of any eligible records, a thorough manual search of the trial list references was conducted.

### Eligibility Criteria

RCTs conducted using the following PICO criteria were included: population (P), patients undergoing thyroidectomy, regardless of the approach (either conventional, endoscopic, or robotic surgery) were eligible; intervention (I), ropivacaine, regardless of the dosing protocol; control (C), placebo or no treatment; and outcomes (O): the primary outcome was pain severity, either assessed by VAS^22^ or numerical rating scale (NRS).^23^ Secondary outcomes included total opioid analgesia consumption, patient satisfaction score, length of hospital stay, postanesthesia care unit (PACU) length of stay, surgery duration, and the incidence of PONV. Furthermore, we excluded quasi‐randomized studies, conference presentations/proceedings, observational studies, in vitro studies, and reviews.

### Study Selection

Two independent reviewers (F.A. and A.A.) performed a comprehensive screening process. After removing duplicate records, each unique record was independently assessed in two stages by the reviewers; this process involved initial title and abstract screening, followed by subsequent full‐text screening of those records that passed the initial phase. Differences were resolved through discussion.

### Data Extraction

To design an Excel extraction form, the full texts of all relevant publications were first obtained; this allowed for a pilot extraction. The form incorporated three sections: included trials' summary characteristics (study ID, country, study design, total patients, treatment protocols, follow‐up duration, infiltration time, surgery type, and pain assessment tool); included participants' baseline characteristics (age and gender); and the outcomes: pain at different follow‐up time points, analgesia consumption, patients' satisfaction, length of hospital stay, PACU length of stay, surgery duration, and the incidence of nausea/vomiting.

Data extraction was independently performed by three reviewers (De.A., H.A., and Da.A.), with any discrepancies subsequently resolved through discussion and consensus with a senior author (E.A.). An event/total format was employed for dichotomous outcomes, whereas means and standard deviations described continuous outcomes. We utilized the formulas given by Wan et al^24^ to convert the data from median and interquartile range or range to mean and standard deviation. Finally, the WebPlotDigitizer online tool was implemented to extract raw data from figures.^25^

### Risk of Bias and Certainty of Evidence

We used the revised Cochrane Collaboration tool for RCTs (ROB 2) to assess the risk of bias in included studies.^26^ Two reviewers (E.A. and F.A.) independently assessed each study, evaluating its selection criteria, performance quality, reporting methods, attrition rates, and overall biases; disagreements were resolved through a consensus‐building process. Also, the certainty of evidence was assessed using the Grading of Recommendations Assessment, Development, and Evaluation (GRADE) framework, which considered factors like inconsistency, imprecision, indirectness, publication bias, and risk of bias.^27^, ^28^ Each factor was individually evaluated, and the decisions were duly justified and documented. Any inconsistencies were resolved through discussion.

### Statistical Analysis

The statistical analysis was performed using Stata MP v. 17 by Stata Corp. We utilized the risk ratio (RR) to combine dichotomous outcomes and mean difference (MD) to combine continuous outcomes, along with a 95% confidence interval (CI). When different assessment tools were reported for the same continuous outcome, we used the standardized MD (SMD). We utilized the fixed‐effects model unless there was significant heterogeneity, in which case we employed the random‐effects model. An assessment of the statistical heterogeneity among the included studies was conducted using the chi‐square test and the *I*‐square statistic (*I* ^2^); we defined statistical significance using a threshold of *P* < .1 for the chi‐square test alongside an *I* ^2^ value of 50% or higher to represent significant heterogeneity. Leave‐one‐out sensitivity analysis and Galbraith plot were conducted in case of significant heterogeneity to investigate the magnitude of each study on the pooled estimate and the potential cause of heterogeneity, respectively. Publication bias was not investigated, as all assessed outcomes had less than 10 RCTs.^29^

## Results

### Search Results and Study Selection

**Figure 1:**
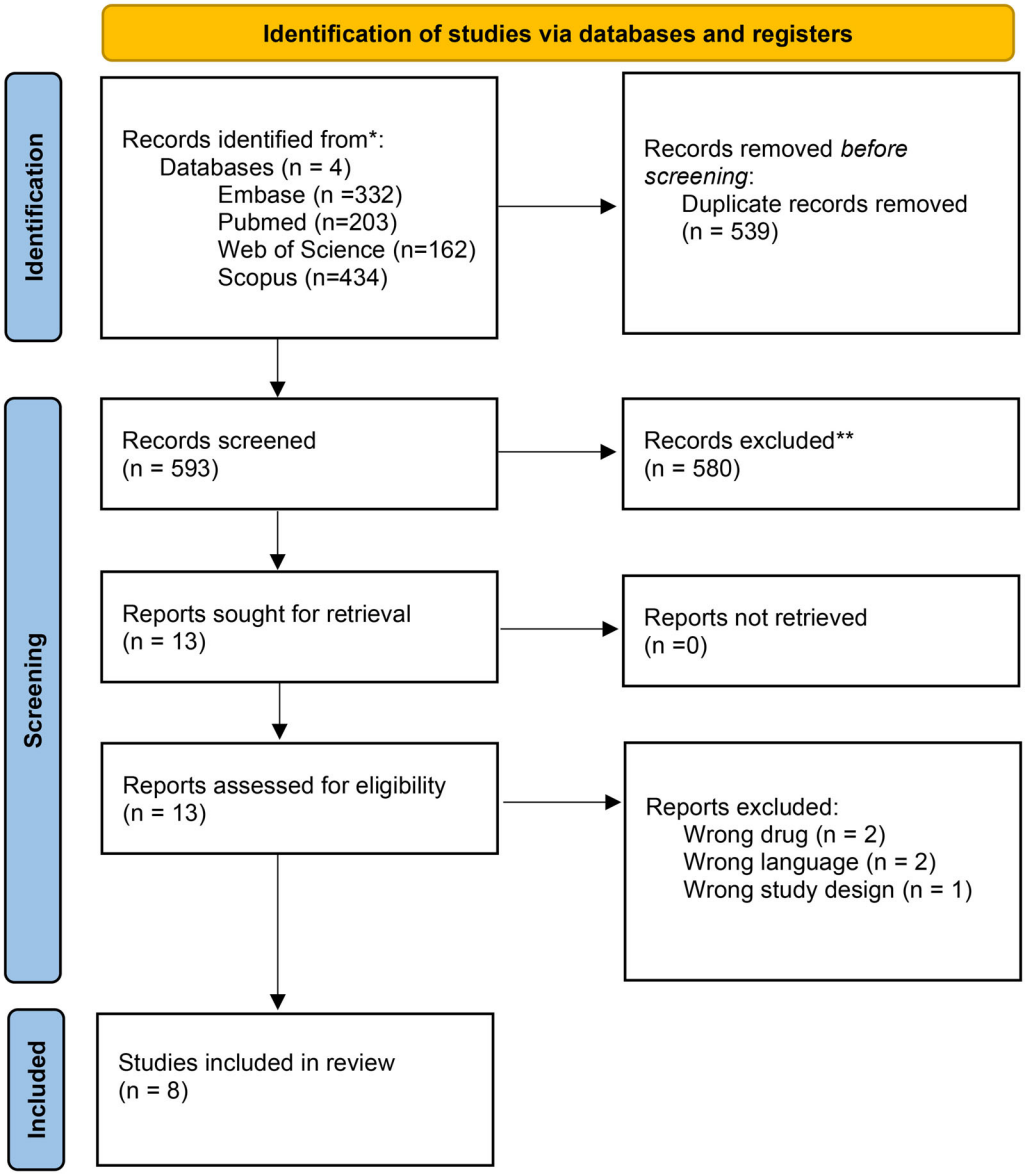
Preferred Reporting Items for Systematic Reviews and Meta‐Analyses flow chart of the screening process.

Following the literature search, 1131 records were identified, and we automatically removed 593 duplicates. Following title and abstract screening, 580 studies failing to meet the inclusion criteria were excluded, leaving 13 full‐text articles for further assessment. Five studies were excluded—which are outlined in Supplemental Table S2, available online—leaving eight RCTs^7^, ^8^, ^12^, ^16^, ^17^, ^18^, ^19^, ^30^ to be included in qualitative and quantitative analysis (Figure 1).

### Characteristics of Included Studies

Eight RCTs and 633 patients were included.^7^, ^8^, ^12^, ^16^, ^17^, ^18^, ^19^, ^30^ Five RCTs included patients undergoing conventional thyroidectomy,^7^, ^8^, ^12^, ^17^, ^19^ two included patients undergoing robotic thyroidectomy,^16^, ^30^ and one included patients undergoing robotic or endoscopic thyroidectomy.^18^ Seven trials used a placebo as a control,^7^, ^8^, ^12^, ^16^, ^18^, ^19^, ^30^ and one trial used no treatment.^17^ Male patients constituted 15.5% (98) of the sample size, whereas females constituted 84.5% (535). Further details on the study design are highlighted in Tables 1 and 2.

**Table 1:** Summary Overview of the Included Randomized Controlled Trials

Study ID	Study design	Country	Duration	Sample size, n	Trial arms		Type of thyroidectomy	Patients criteria
					Intervention	Control		
Ayman et al 2012^17^	RCT	Italy	Not reported	40	Ropivacaine	No infiltration	Conventional	Total thyroidectomy
Bae et al 2016^30^	RCT	South Korea	March 2012 to December 2012	103	Ropivacaine	Placebo	BABA‐RT	Lobectomy or total thyroidectomy
Kang et al 2015^16^	RCT	South Korea	December 2012 to July 2013	34	Ropivacaine	Placebo	BABA‐RT	Lobectomy or total thyroidectomy
Karamanlioglu et al 2005^19^	RCT	Turkey	Not reported	40	Ropivacaine	Placebo	Conventional	Lobectomy or total thyroidectomy
Laskou et al 2024^12^	RCT	Greece	January 2022 to January 2024	45	Ropivacaine	Placebo	Conventional	Thyroidectomy/parathyroidectomy
Lee et al 2017^18^	RCT	South Korea	January 2014 to May 2016	148	Ropivacaine	Placebo	BABA‐RT or BABA‐ET	Lobectomy or total thyroidectomy
Miu et al 2016^8^	RCT	France	October 2009 to April 2010	173	Ropivacaine	Placebo	Conventional	NA
Motamed et al 2007^7^	RCT	France	Not reported	50	Ropivacaine	Placebo	Conventional	Lobectomy or total thyroidectomy

Abbreviations: BABA‐ET, bilateral axillo‐breast approach endoscopic thyroidectomy; BABA‐RT, bilateral axillo‐breast approach robotic thyroidectomy; n, number of patients; NA, not available; RCT, randomized controlled trials.

**Table 2:** Baseline Characteristics of the Included Participants

Study ID	Group	Sample size, n	Dose	Age, y	Sex, n [male/female]	Follow‐up, h	Infiltration timing	Analgesic consumption measurement	Pain tool
Ayman et al 2012^17^	Ropivacaine	20	10 mL of 0.75% ropivacaine	49.3 ± 0.39	[6/14]	24	Before skin incision	Not reported	10‐Point VAS
	Control	20	No infiltration	51.5	[1/19]				
Bae et al 2016^30^	Ropivacaine	53	40 mL of 0.25% ropivacaine	40.5 ± 9.4	[4/49]	24	Before skin closure	0.8 mg/kg intravenous fentanyl was injected slowly with 20 mL of normal saline	10‐Point VAS
	Placebo	50	0.9% of normal saline	39.9 ± 11.1	[3/47]				
Kang et al 2015^16^	Ropivacaine	17	3 mL/kg of 0.1% saline mixed ropivacaine (dose: 3 mg/kg)	38 ± 10.1	[0/17]	66	Before skin incision	Intravenous injection of 50‐mg fentanyl as rescue analgesia	100‐Point VAS
	Placebo	17	0.9% of normal saline	34.7 ± 7.6	[0/17]				
Karamanlioglu et al 2005^19^	Ropivacaine	20	10 mL of 0.75% ropivacaine	51 ± 16	[6/14]	24	Before skin closure	Pethidine 1 mg kg^–1^ intramuscular injection	10‐Point VAS
	Placebo	20	12 mL of normal saline	48 ± 15	[5/15]				
Laskou et al 2024^12^	Ropivacaine	24	15 mL of 100 mg ropivacaine	54.78 ± 15.08	[4/20]	24	Before skin closure	40 mg parecoxib or 50 to 100 mg tramadol was administered. Total analgesic requirements up to 24 h postoperatively were calculated in oral morphine equivalents	10‐Point VAS
	Placebo	21	15 mL of normal saline	54.78 ± 15.08	[5/16]				
Lee et al 2017^18^	Ropivacaine	74	225 mg of ropivacaine and epinephrine 1 mg/1 mL diluted by 1:100,000 that was mixed with 100 mL of normal saline	35.5 ± 8.8	[8/66]	48	During flap raising	Not reported	10‐Point NRS
	Placebo	74	100 mL of normal saline	38.0 ± 9.3	[5/69]				
Miu et al 2016^8^	Ropivacaine	88	10 mL of 0.75% ropivacaine	46 ± 15	[17/71]	24	Before skin closure	Intravenous morphine was titrated every 5 min in 2‐mg increments (3 mg if weight >60 kg)	100‐Point VAS
	Placebo	85	10 mL of 0.9% normal saline	48 ± 14	[22/63]				
Motamed et al 2007^7^	Ropivacaine	25	2% of ropivacaine	41 ± 6	[7/18]	24	Before skin closure	Subcutaneous morphine (5‐10 mg)	100‐Point VAS
	Placebo	25	Normal saline	43 ± 8	[5/20]				

Abbreviations: n, number of patients; NRS, numerical rating scale; VAS, visual analog scale.

### Risk of Bias and Certainty of Evidence

**Figure 2:**
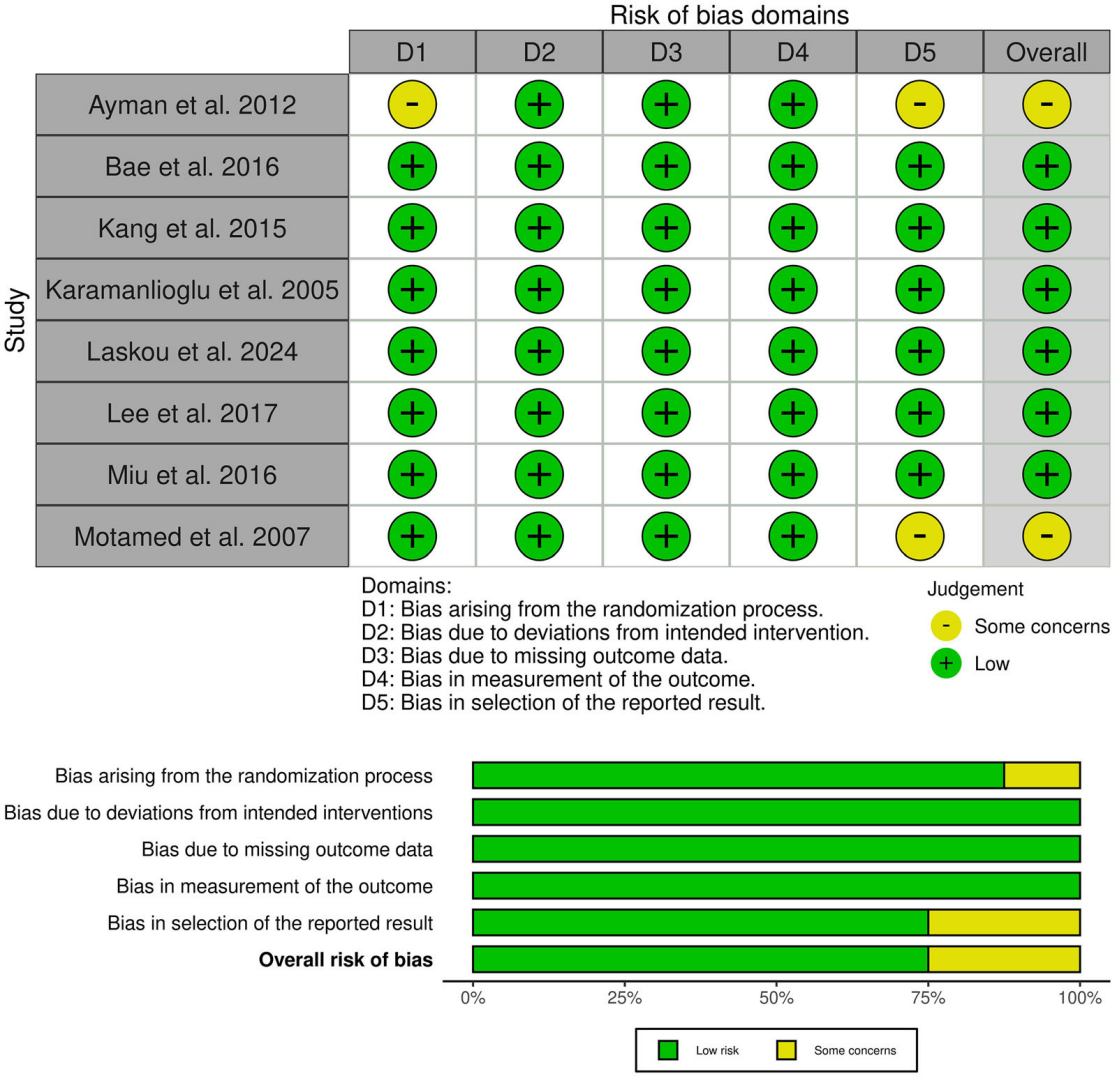
Quality assessment of risk of bias in the included trials. The upper panel presents a schematic representation of risks (low = red, some concern = yellow, and high = red) for specific types of biases of the studies in the review. The lower panel presents risks (low = red, some concern = yellow, and high = red) for the subtypes of biases of the combination of studies included in this review.

Six out of eight RCTs showed a low overall risk of bias, as depicted in Figure 2. Ayman et al showed some concerns of selection bias due to the absence of details on randomization and allocation processes.^17^ Ayman et al and Motamed et al showed some concerns of reporting bias due to the lack of a registered protocol.^7^, ^17^ Also, the details of the certainty of evidence are outlined in Table 3.

**Table 3:** Grading of Recommendations Assessment, Development, and Evaluation Evidence Profile

Certainty assessment							Summary of findings				
Participants (studies) Follow‐up	Risk of bias	Inconsistency	Indirectness	Imprecision	Publication bias	Overall certainty of evidence	Study event rates, %		Relative effect (95% CI)	Anticipated absolute effects	
							With [control]	With [ropivacaine]		Risk with [control]	Risk difference with [ropivacaine]
Pain after 1‐2 h											
365 (5 RCTs)	Not serious	Very serious^a^	Not serious	Serious^b^	None	⊕◯◯◯ Very low^a^^,^ b	181	184	‐	‐	SMD 1.4 SD lower (2.3 lower to 0.51 lower)
Pain after 4 h											
504 (5 RCTs)	Not serious	Very serious^a^	Not serious	Serious^c^	None	⊕◯◯◯ Very low^a^^,^ c	249	255	‐	‐	SMD 0.35 SD lower (0.79 lower to 0.08 higher)
Pain after 6‐8 h											
538 (6 RCTs)	Not serious	Very serious^a^	Not serious	Serious^c^	None	⊕◯◯◯ Very low^a^^,^ c	266	272	‐	‐	SMD 0.61 SD lower (1.22 lower to 0)
Pain after 16‐18 h											
390 (5 RCTs)	Not serious	Very serious^a^	Not serious	Very serious^b^^,^ c	None	⊕◯◯◯ Very low^a^^,^ b ^,^ c	192	198	‐	‐	SMD 0.61 SD lower (1.32 lower to 0.11 higher)
Pain after 24 h											
464 (4 RCTs)	Not serious	Not serious	Not serious	Not serious	None	⊕⊕⊕⊕ High	229	235	‐	‐	SMD 0.08 SD lower (0.26 lower to 0.1 higher)
Analgesia consumption											
445 (6 RCTs)	Not serious	Very serious^a^	Not serious	Serious^c^	None	⊕◯◯◯ Very low^a^^,^ c	218	227	‐	‐	SMD 0.75 SD lower (1.3 lower to 0.2 lower)
Surgery duration											
460 (7 RCTs)	Not serious	Serious^d^	Not serious	Serious^c^	None	⊕⊕◯◯ Low^c^^,^ d	227	233	‐	227	MD 1.61 min lower (7.47 lower to 4.25 higher)
PACU length of stay											
223 (2 RCTs)	Not serious	Very serious^a^	Not serious	Extremely serious^b^^,^ c	None	⊕◯◯◯ Very low^a^^,^ b ^,^ c	110	113	‐	110	MD 21.08 h higher (56.87 lower to 14.71 higher)
Length of hospital stay											
177 (3 RCTs)	Not serious	Not serious	Not serious	Serious^b^	None	⊕⊕⊕◯ Moderate^b^	87	90	‐	87	MD 0.06 d lower (0.16 lower to 0.05 higher)
Patients' satisfaction											
268 (3 RCTs)	Not serious	Not serious	Not serious	Serious^b^	None	⊕⊕⊕◯ Moderate^b^	131	137	‐	‐	SMD 0.14 SD higher (0.1 lower to 0.38 higher)
Postoperative nausea and vomiting											
593 (7 RCTs)	Not serious	Not serious	Not serious	Not serious	None	⊕⊕⊕⊕ High	45/292 (15.4%)	48/301 (15.9%)	RR 1.01 (0.70‐1.45)	45/292 (15.4%)	2 more per 1000 (from 46 fewer to 69 more)

Abbreviations: CI, confidence interval; MD, mean difference; PACU, postanesthesia care unit; RCT, randomized controlled trial; RR, risk ratio; SMD, standardized mean difference.

Explanations:

^a^ *I* ^2^ > 75%.

^b^ Low number of participants.

^c^ A wide confidence interval, not excluding the appreciable harm or benefit.

^d^ *I* ^2^ > 50%.

### Primary Outcome: Pain Severity

**Figure 3:**
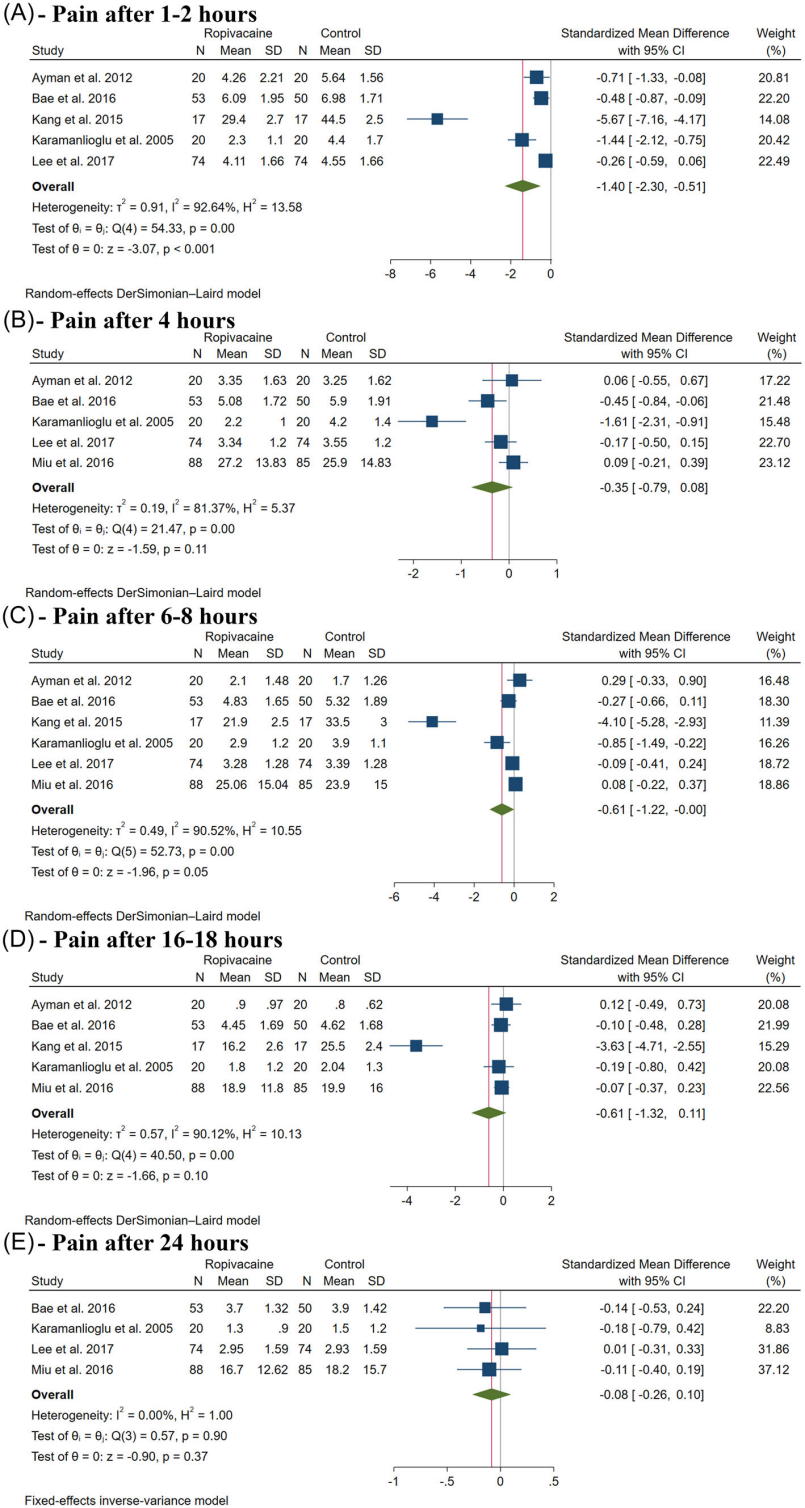
Forest plot of the primary outcome (pain). CI, confidence interval.

Ropivacaine significantly decreased pain after 1 to 2 hours postoperatively (SMD: −1.40, with 95% CI [−2.30, −0.51], *P* < .001, *I* ^2^ = 93%, Figure 3A). However, there was no difference between both groups after 4 hours (SMD: −0.35, with 95% CI [−0.79, 0.08], *P* = .11, *I* ^2^ = 81%, Figure 3B), 6 to 8 hours (SMD: −0.61, with 95% CI [−1.22, −0.00], *P* = .05, *I* ^2^ = 91%, Figure 3C), 16 to 18 hours (SMD: −0.61, with 95% CI [−1.32, 0.11], *P* = .10, *I* ^2^ = 90%, Figure 3D), and 24 hours (SMD: −0.08, with 95% CI [−0.26, 0.10], *P* = .37, *I* ^2^ = 0%, Figure 3E).

Leave‐one‐out sensitivity analysis showed consistent results in pain after 1 to 2 hours (Supplemental Figure S1, available online), 4 hours (Supplemental Figure S2, available online), and 16 to 18 hours (Supplemental Figure S3, available online). Still, ropivacaine significantly decreased pain after 6 to 8 hours after excluding Ayman et al (*P* = .024) and Miu et al (*P* = .045) (Supplemental Figure S4, available online). Galbraith plots showed that Kang et al is a potential outlier and potentially responsible for the observed heterogeneity in pain after 1 to 2 hours (Supplemental Figure S5, available online), 6 to 8 hours (Supplemental Figure S7, available online), and 16 to 18 hours (Supplemental Figure S8, available online), but Karamanlioglu et al was the most likely responsible for heterogeneity in pain after 4 hours (Supplemental Figure S6, available online).

The test for subgroup analysis based on thyroidectomy type was significant in pain after 1 to 2 hours (*P* = .08) (Supplemental Figure S9, available online); however, it was not significant after 4 hours (*P* = .54) (Supplemental Figure S10, available online), 6 to 8 hours (*P* = .56) (Supplemental Figure S11, available online), 16 to 18 hours (*P* = .32) (Supplemental Figure S12, available online), and 24 hours (*P* = .77) (Supplemental Figure S13, available online).

### Secondary Outcomes

#### Total Opioid Analgesia Consumption

**Figure 4:**
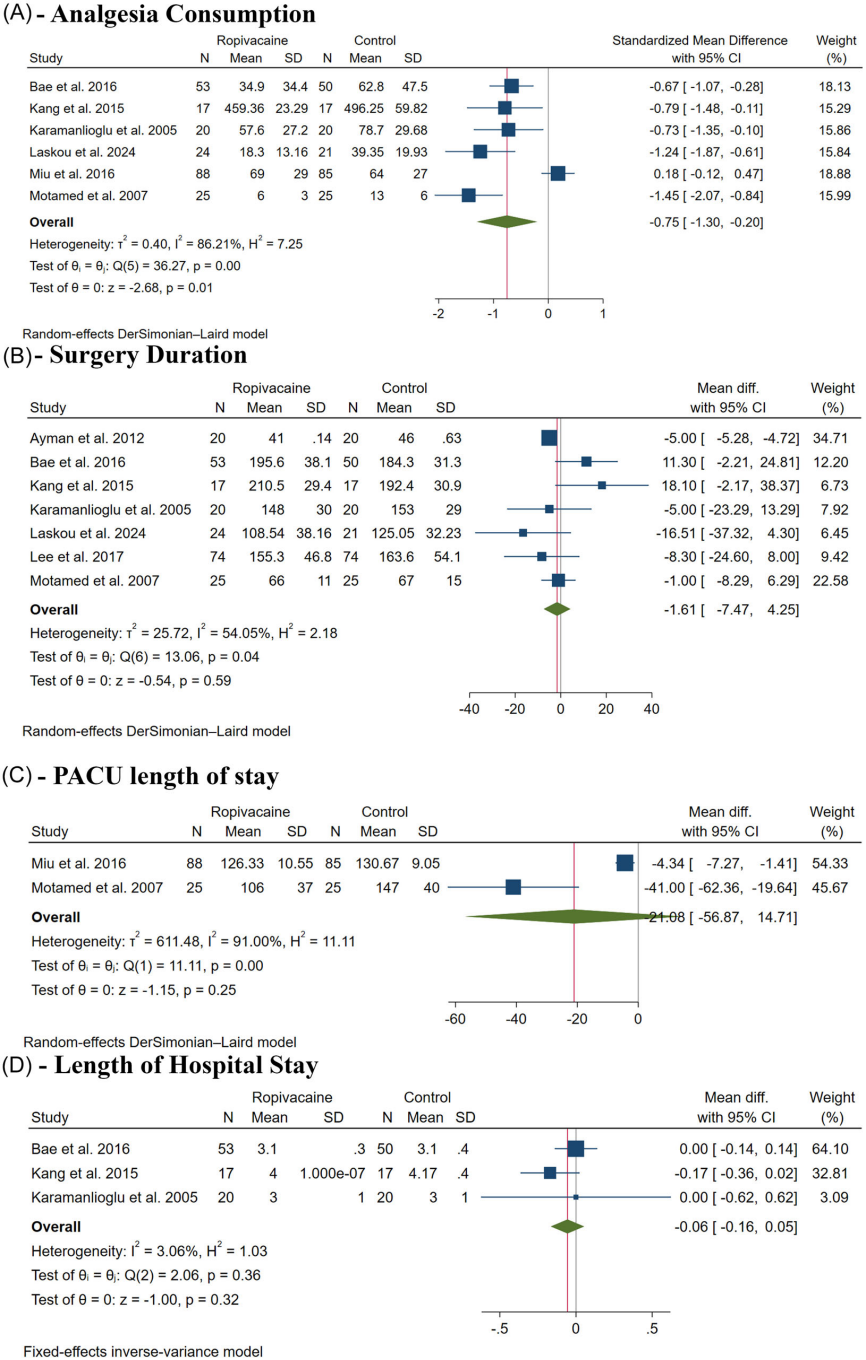
Forest plot of the secondary outcomes. CI, confidence interval; PACU, postanesthesia care unit.

Ropivacaine significantly decreased total opioid analgesia consumption (SMD: −0.75, with 95% CI [−1.30, −0.20], *P* = .01, *I* ^2^ = 86%, Figure 4A). Leave‐one‐out sensitivity analysis showed consistent results (Supplemental Figure S14, available online). The Galbraith plot showed that Laskou et al, Miu et al, and Motamed et al are potential outliers and potentially responsible for the observed heterogeneity (Supplemental Figure S15, available online). The test for subgroup analysis based on thyroidectomy type was insignificant (*P* = .86) (Supplemental Figure S16, available online).

#### Surgery Duration and Hospitalization

There was no difference between both groups in surgery duration (MD: −1.61 minutes, with 95% CI [−7.47, 4.25], *P* = .59, *I* ^2^ = 54%, Figure 4B), PACU length of stay (MD: 21.08 hours, with 95% CI [−56.87, 14.71], *P* = .25, *I* ^2^ = 91%, Figure 4C), and length of hospital stay (MD: −0.06 days, with 95% CI [−0.16, 0.05], *P* = .32, *I* ^2^ = 3%, Figure 4D).

Leave‐one‐out sensitivity analysis showed consistent results in surgery duration (Supplemental Figure S17, available online). The Galbraith plot showed that Ayman et al is a potential outlier and potentially responsible for the observed heterogeneity in surgery duration (Supplemental Figure S18, available online). The test for subgroup analysis based on thyroidectomy type was significant in surgery duration (*P* = .01), showing that ropivacaine significantly decreased conventional thyroidectomy duration but increased robotic thyroidectomy duration (Supplemental Figure S19, available online).

#### Patients' Satisfaction

**Figure 5:**
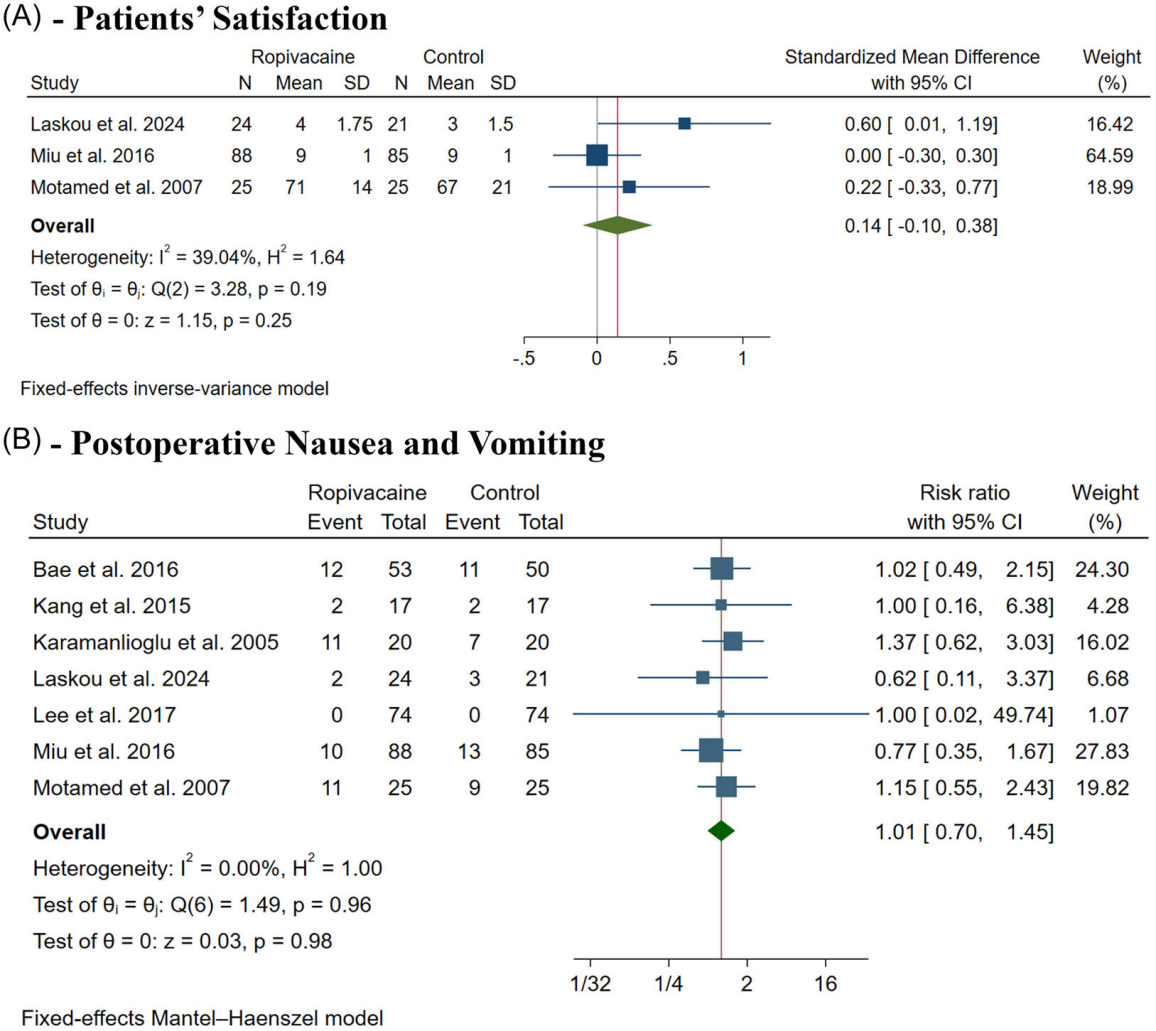
Forest plot of the secondary outcomes. CI, confidence interval.

Patients' satisfaction was assessed in three trials.^7^, ^8^, ^12^ Laskou et al evaluated satisfaction with a 5‐point scale upon discharge.^12^ Miu et al asked the patients to assess their global satisfaction about pain management based on a numeric scale ranging from 0 to 10.^8^ Finally, Motamed et al assessed patients' satisfaction, using 0 to 100 VAS.^7^ There was no difference between both groups (SMD: −1.14, with 95% CI [−0.01, 0.38], *P* = .25, *I* ^2^ = 39%, Figure 5A).

#### Postoperative Nausea and Vomiting

There was no difference between both groups (RR: 1.01, with 95% CI [0.70, 1.45], *P* = .98, *I* ^2^ = 0%, Figure 5B). The test for subgroup analysis based on thyroidectomy type was insignificant (*P* = 1.00) (Supplemental Figure S20, available online).

## Discussion

Pooling evidence from eight RCTs and 633 patients showed that local ropivacaine infiltration reduced postthyroidectomy pain for up to 1 to 2 hours, with no significant difference between ropivacaine and control at 4, 6 to 8, 16 to 18, and 24 hours. Additionally, ropivacaine was associated with a statistically significant reduction in postoperative analgesic consumption compared to the control group. However, there was no significant difference between the two groups regarding surgery duration, PACU stay, hospital stay, and patient satisfaction. Finally, ropivacaine was well tolerated, with comparable PONV rates to control.

The pooled analysis, particularly for pain severity outcomes, revealed a considerable degree of heterogeneity. This variability may be attributed to several factors, including the use of different pain assessment tools, variations in patient characteristics (ie, total thyroidectomy vs lobectomy), differences in surgical techniques (conventional vs robotic thyroidectomy), and the administration of varying doses and protocols of ropivacaine. To account for these differences and minimize the influence of confounding variables unrelated to ropivacaine infiltration, we employed the SMD as the effect measure. This method, as recommended by the Cochrane Collaboration when outcomes are reported using different scales, allowed for the standardization of pain scores across studies and helped isolate the effect of ropivacaine infiltration.^21^ Moreover, the use of SMD contributed to the overall robustness and methodological rigor of the analysis.

Multiple factors contribute to postoperative discomfort following thyroid surgery. Postoperative incisional pain, particularly on the first day, is a significant source of patient discomfort.^8^ The challenges of complex surgical techniques, painful swallowing secondary to endotracheal intubation, and neck discomfort from hyperextension are further contributing factors.^31^ Effective implementation of a structured analgesic protocol is paramount to achieving the goals of Enhanced Recovery After Surgery (ERAS) protocols: shorter hospital stays and faster functional recovery for patients.^32^

Given that the thyroidectomy site is primarily innervated by superficial cervical plexus branches, regional anesthesia techniques, including local anesthesia and cervical plexus blocks, may offer a promising approach.^12^ Because it is both a safe and feasible procedure, local wound infiltration provides considerable advantages. The infiltration of the thyroidectomy wound appears to lengthen the duration before patients receive rescue analgesia, suggesting a correlation between surgical site infiltration and pain relief.^13^, ^33^ Thyroidectomy wound infiltration commonly utilizes the amide local anesthetics bupivacaine, lidocaine, and ropivacaine.^8^

Ropivacaine successfully ameliorated postoperative pain but only for up to 1 to 2 hours. Postthyroidectomy pain increases for up to 3 hours postoperatively, after which it starts to decline, regardless of the local analgesia.^32^ Postthyroidectomy pain peaks within the first hour, subsequently diminishing after 3 hours, becoming minimal and easily manageable with simple analgesia at 8 hours, and almost nonexistent by 16 hours postoperatively.^17^ Also, the duration of ropivacaine's action, which ranges from 2 to 6 hours, may partly explain the faded effect after 2 hours postoperatively.^30^ Notably, in our subgroup analysis, this effect persisted for conventional open surgery but not for robotic surgery. Individuals undergoing minimally invasive robotic thyroidectomy report higher rates of immediate postoperative pain compared to those undergoing open procedures.^34^, ^35^ Although the early reduction in pain may offer some initial comfort and potentially reduce the need for rescue analgesia in the immediate postoperative period, the clinical relevance of this short‐lived benefit remains limited. It is unlikely to impact overall patient recovery, hospital stay, or long‐term outcomes. Therefore, although statistically significant, the short‐term analgesic effect of ropivacaine should be interpreted with caution, particularly when considering its role in standard postoperative pain management protocols. Although this limited duration of effect may appear inconsistent with the relatively long half‐life of ropivacaine, it is important to note that the timing of administration varied across the included studies. In some trials, ropivacaine was infiltrated before skin incision or during flap elevation, whereas in others it was administered before skin closure. These variations may have contributed to differences in the onset and duration of analgesia observed in the early postoperative phase.

Postoperative pain following skin flap elevation in robotic thyroidectomy may be more intense than that experienced after conventional open thyroidectomy.^30^, ^36^ The bilateral axillo‐breast approach (BABA), in particular, requires extensive subcutaneous chest flap dissection to achieve adequate surgical exposure via bilateral axillary and breast incisions.^30^ In addition, the use of carbon dioxide insufflation during BABA can cause skin flap tension, potentially contributing to increased discomfort.^16^ These technical differences in surgical access, extent of tissue manipulation, and neural irritation may significantly influence postoperative pain profiles. In the present meta‐analysis, subgroup analysis based on surgical approach revealed that early postoperative pain scores (1‐4 hours) differed between robotic and conventional thyroidectomy, suggesting a potential confounding effect on the overall pooled estimate. However, this inconsistency may not translate into a clinically meaningful difference, particularly given the small effect size. In contrast, late postoperative pain scores (6‐24 hours) were consistent and comparable across both surgical approaches. Nonetheless, these findings should be interpreted with caution in light of the aforementioned limitations.

Furthermore, this effect on short‐term pain alleviation was reflected in analgesia consumption, significantly decreasing the need for analgesia compared to control. Although patient‐controlled analgesia can optimize analgesic efficacy when commenced with pain onset, continuous systemic narcotic infusion is often associated with adverse events such as respiratory depression, excessive sedation, pruritus, constipation, ileus, and PONV.^37^ Hence, using ropivacaine routinely without contraindication after thyroidectomy, regardless of its type, is a promising strategy to enhance postoperative outcomes. In the present meta‐analysis, most of the included RCTs assessed total postoperative analgesic consumption in cases of severe, intolerable pain. However, there was significant variation in the type of opioids used (eg, morphine, pethidine), the units of measurement, and the administered doses. Due to this heterogeneity, we standardized the effect size using the SMD, following the Cochrane Collaboration guidelines.^21^ Although the pooled estimate (SMD = –0.75) suggests a moderate reduction in opioid use, interpreting its true clinical significance remains challenging. Further well‐designed RCTs with consistent opioid reporting—preferably in morphine milliequivalents—are needed to determine whether this reduction is indeed clinically meaningful.

Moreover, ropivacaine was well‐tolerated, without any increase in PONV rates. Decreasing analgesia consumption, without any increase in PONV, is a significant advantage. Also, Bae et al reported that the ropivacaine group exhibited no signs of drug hypersensitivity, hypotension, or hepatic or cardiovascular adverse events.^30^ Additionally, our results showed it had no effect on surgery duration.^30^ The findings show that ropivacaine administration is safe for patients undergoing thyroidectomy. Nevertheless, some patients may need special consideration, including those with age‐related issues, arrhythmias, hypotension, moderate to severe liver or kidney problems, or allergies to anesthetics.^30^

The generalizability of our findings should be considered in the context of the health care systems represented in the included studies (eg, South Korea, France, and Italy), all of which are high‐resource settings with standardized postoperative care protocols. In resource‐limited regions, where access to advanced analgesics (eg, patient‐controlled analgesia, rescue opioids) or monitoring facilities (eg, PACU) may be inconsistent, ropivacaine infiltration could serve as a cost‐effective and logistically feasible option to improve postoperative pain management. Its low cost, long shelf life, and minimal infrastructure requirements (eg, no need for continuous monitoring) make it particularly suitable in such settings. Conversely, in well‐resourced health care systems, where multimodal analgesia and opioid‐sparing strategies are routinely employed, the clinical utility of ropivacaine may lie in its ability to further reduce early opioid consumption and related side effects (eg, PONV, respiratory depression). Future research should evaluate the efficacy and implementation of ropivacaine in resource‐constrained environments, where its simplicity and affordability could help address critical gaps in postoperative care.

A few limitations constrain our findings. First, we included eight RCTs; however, the sample size remains relatively small compared to the number of thyroidectomy patients. Second, the included trials had different dosing protocols of ropivacaine, which may be responsible for the observed heterogeneity in the treatment effect. Third, all included trials were conducted in resourceful countries with well‐established health care systems; thus, our findings may have limited generalizability over resource‐deficient centers. Fourth, the assessment and reporting methods for pain, analgesia consumption, and patient satisfaction varied among the included trials, but we employed SMD to mitigate this variation. Fifth, we could not assess the effect of additional interventions, such as epinephrine infusion, on the reported clinical outcomes. Finally, the GRADE assessment of our findings showed low to very low certainty of evidence, reflecting the heterogeneity and imprecision in the current evidence; therefore, our findings should be interpreted with caution.

## Conclusion

Ropivacaine local infiltration after thyroidectomy significantly decreased pain for up to 1 to 2 hours and reduced analgesic consumption compared to control, although the certainty of evidence remains low. However, ropivacaine had no significant effect on pain between 4 and 24 hours, surgery duration, PACU stay, hospital stay, or patient satisfaction. It was well‐tolerated, with a comparable rate of PONV to control. The clinical benefit of ropivacaine local infiltration remains limited, and further large‐scale, high‐quality RCTs are needed to better define its role—particularly in resource‐limited settings where cost‐effective pain management strategies are essential.

## Author Contributions

Ebraheem Albazee contributed to study conception, study design, data collection, data analysis, write‐up of original draft of manuscript, and review of manuscript for editorial and intellectual contents. Fahad Allafi, Abdulwahab Alsalem, Deemah AlShaya, Hayfaa Alhazami, and Danah Alfalah contributed to literature review, data collection, and review of manuscript for editorial and intellectual contents. All authors read and approved the final draft of manuscript.

## Disclosures

### Competing interests

None.

### Funding source

None.

## Supporting information

Supplemental_04‐07‐2025.

## Data Availability

All data are available within the manuscript and can be obtained from the corresponding author upon a reasonable request.
