# Supplementary material for: Ropivacaine Local Infiltration for Pain Control After Thyroidectomy: A Systematic Review and Meta‐Analysis
Source: OTO Open. 2025 May 5;9(2):e70124. doi: 10.1002/oto2.70124 (PMC12051372; doi:10.1002/oto2.70124)
Supplement: Supplementary file 1 — Supplemental_04‐07‐2025. [file OTO2-9-e70124-s001.docx]

| **Table S1.** The exact search strategy for each database. | | | |
| --- | --- | --- | --- |
| **Database** | **Search Field** | **Search strategy** | **Results** |
| **PubMed** | All Fields | (thyroidectom* OR “total thyroidectomy” OR “thyroid surgery”) AND (ropivacain* OR “ropivacaine hydrochloride” OR “ropivacaine monohydrochloride” OR naropeine OR naropin OR “LEA 103” OR “LEA-103” OR “AL 381” OR “1 Propyl 2',6' pipecoloxylidide” OR “(S)-Ropivacaine” OR “84057-95-4” OR “rocaine” OR “local anaesthesia” OR “local anesthesia” OR “local analgesia” OR “local anesthetic” OR “local anaesthetic”) | 203 |
| **CENTRAL** | Title Abstract Keyword | (thyroidectom* OR “total thyroidectomy” OR “thyroid surgery”) AND (ropivacain* OR “ropivacaine hydrochloride” OR “ropivacaine monohydrochloride” OR naropeine OR naropin OR “LEA 103” OR “LEA-103” OR “AL 381” OR “1 Propyl 2',6' pipecoloxylidide” OR “(S)-Ropivacaine” OR “84057-95-4” OR “rocaine” OR “local anaesthesia” OR “local anesthesia” OR “local analgesia” OR “local anesthetic” OR “local anaesthetic”) | 130 |
| **Web of Science** | All Fields | (thyroidectom* OR “total thyroidectomy” OR “thyroid surgery”) AND (ropivacain* OR “ropivacaine hydrochloride” OR “ropivacaine monohydrochloride” OR naropeine OR naropin OR “LEA 103” OR “LEA-103” OR “AL 381” OR “1 Propyl 2',6' pipecoloxylidide” OR “(S)-Ropivacaine” OR “84057-95-4” OR “rocaine” OR “local anaesthesia” OR “local anesthesia” OR “local analgesia” OR “local anesthetic” OR “local anaesthetic”) | 162 |
| **Scopus** | Article title, Abstract, Keywords | (thyroidectom* OR “total thyroidectomy” OR “thyroid surgery”) AND (ropivacain* OR “ropivacaine hydrochloride” OR “ropivacaine monohydrochloride” OR naropeine OR naropin OR “LEA 103” OR “LEA-103” OR “AL 381” OR “1 Propyl 2',6' pipecoloxylidide” OR “(S)-Ropivacaine” OR “84057-95-4” OR “rocaine” OR “local anaesthesia” OR “local anesthesia” OR “local analgesia” OR “local anesthetic” OR “local anaesthetic”) | 434 |
| **Google Scholar** | All Fields | (“thyroid surgery”) AND (ropivacaine) | 709 |

| **Study ID** | **Title** | **Reason for Exclusion** |
| --- | --- | --- |
| **Materazzi et al.** | Efficacy on pain of Pubivacaine versus Ropivacaine infiltration before thyroidectomy: Prospective randomized study | Not in English |
| **Balga et al.** | Does a second local anesthesia infiltration in thyroid reduce early and late nociceptive pain? | Wrong drug (not ropivacaine) |
| **Lin et al.** | Addition of dexmedetomidine to ropivacaine improves cervical plexus block | Wrong drug + wrong outcome |
| **Dhillon et al.** | Perioperative analgesia for thyroid and parathyroid surgery: a review of current practices | Wrong study design |
| **Miu et al.** | Efficacy of preoperative scar infiltration by ropivacaine in thyroid surgery: a prospective randomized study | Not in English |

**Table S2**. Excluded records during full-text screen.


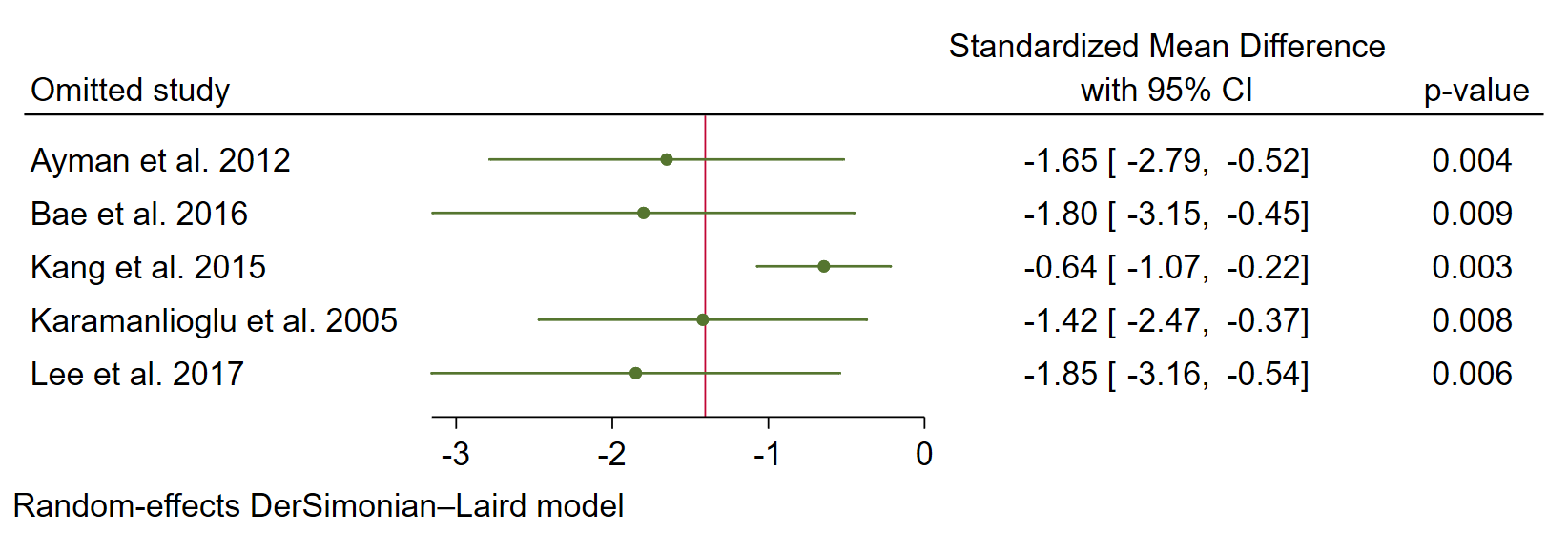


**Figure S1.** Leave-one-out sensitivity analysis of pain after 1-2 hours.


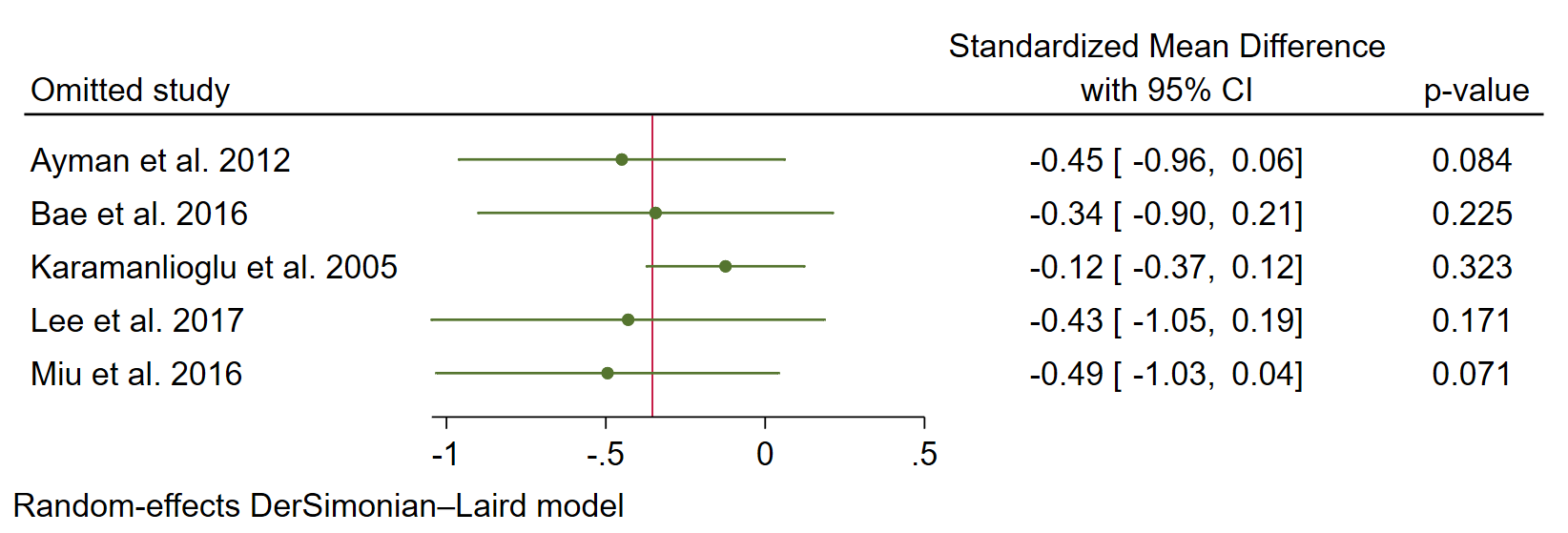


**Figure S2.** Leave-one-out sensitivity analysis of pain after 4 hours.


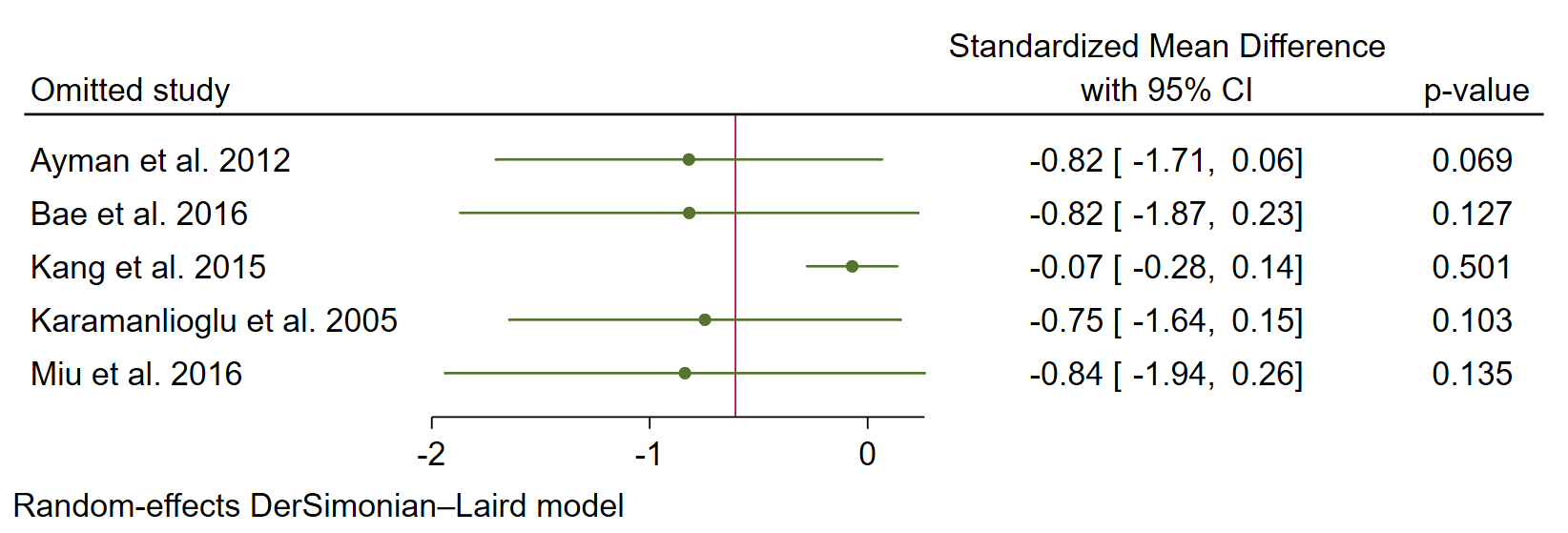


**Figure S3.** Leave-one-out sensitivity analysis of pain after 16-18 hours.


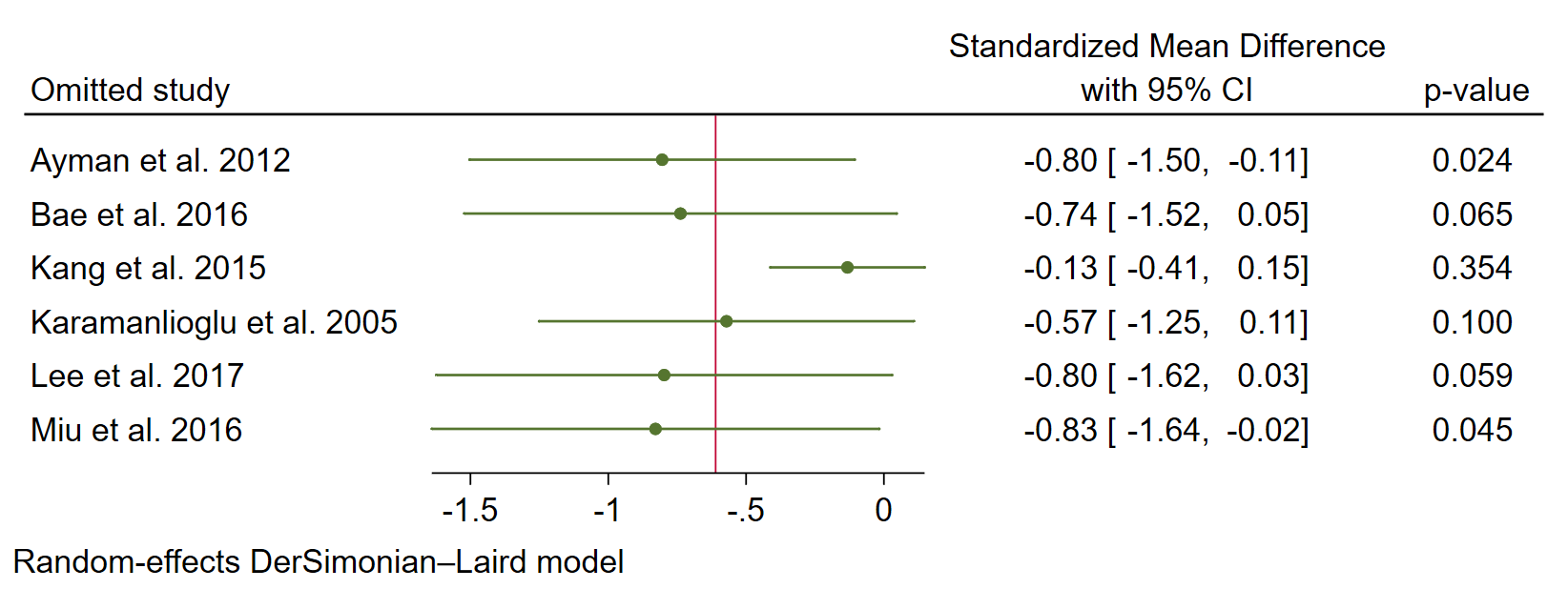


**Figure S4.** Leave-one-out sensitivity analysis of pain after 6-8 hours.


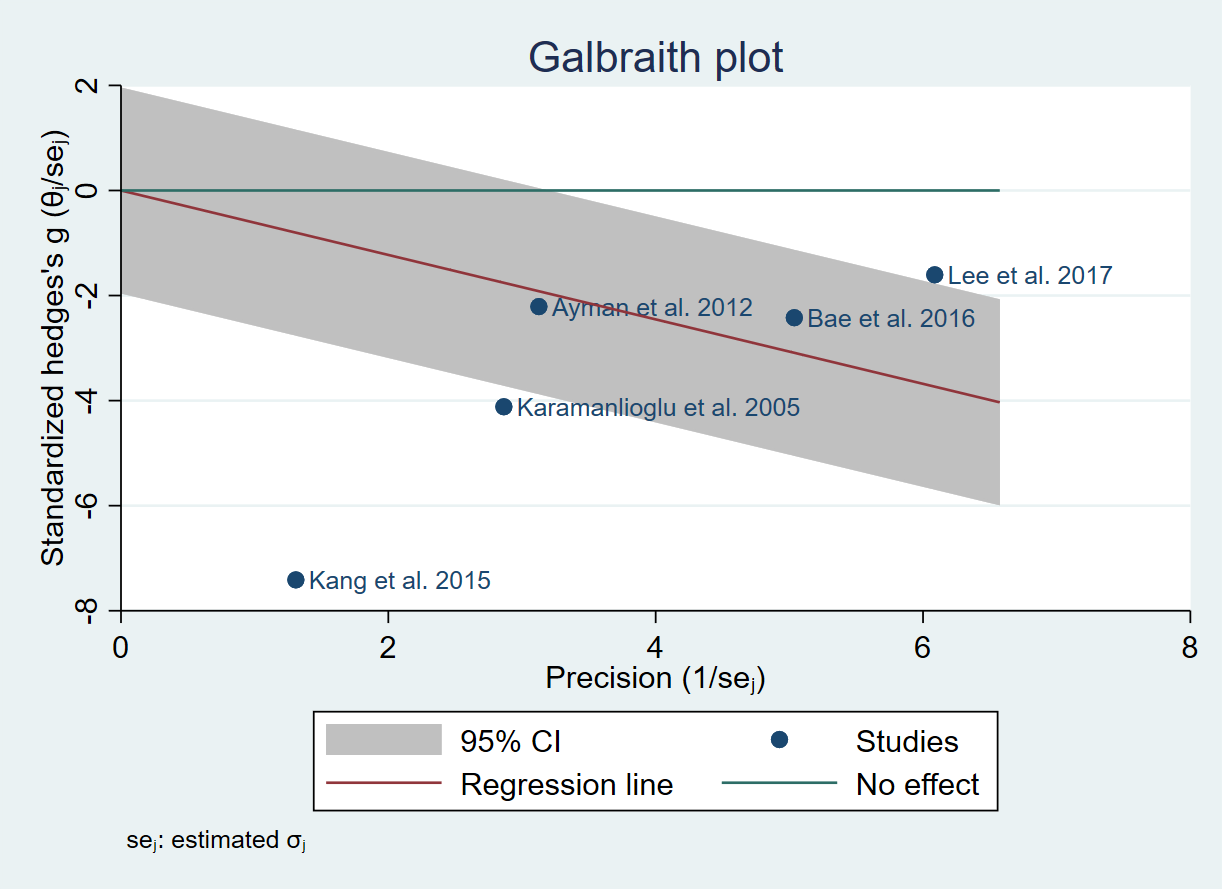


**Figure S5**. Galbraith plot of pain after 1-2 hours.


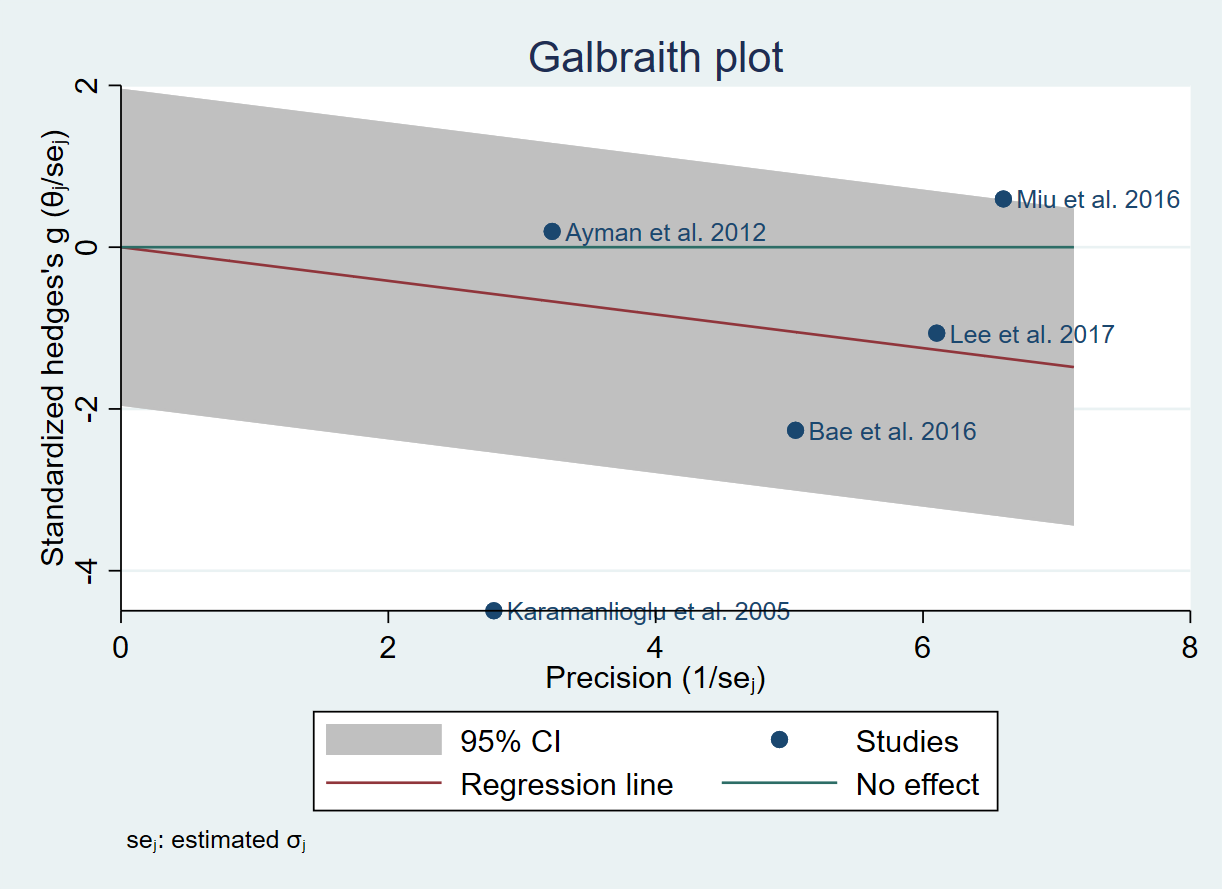


**Figure S6.** Galbraith plot of pain after 4 hours.


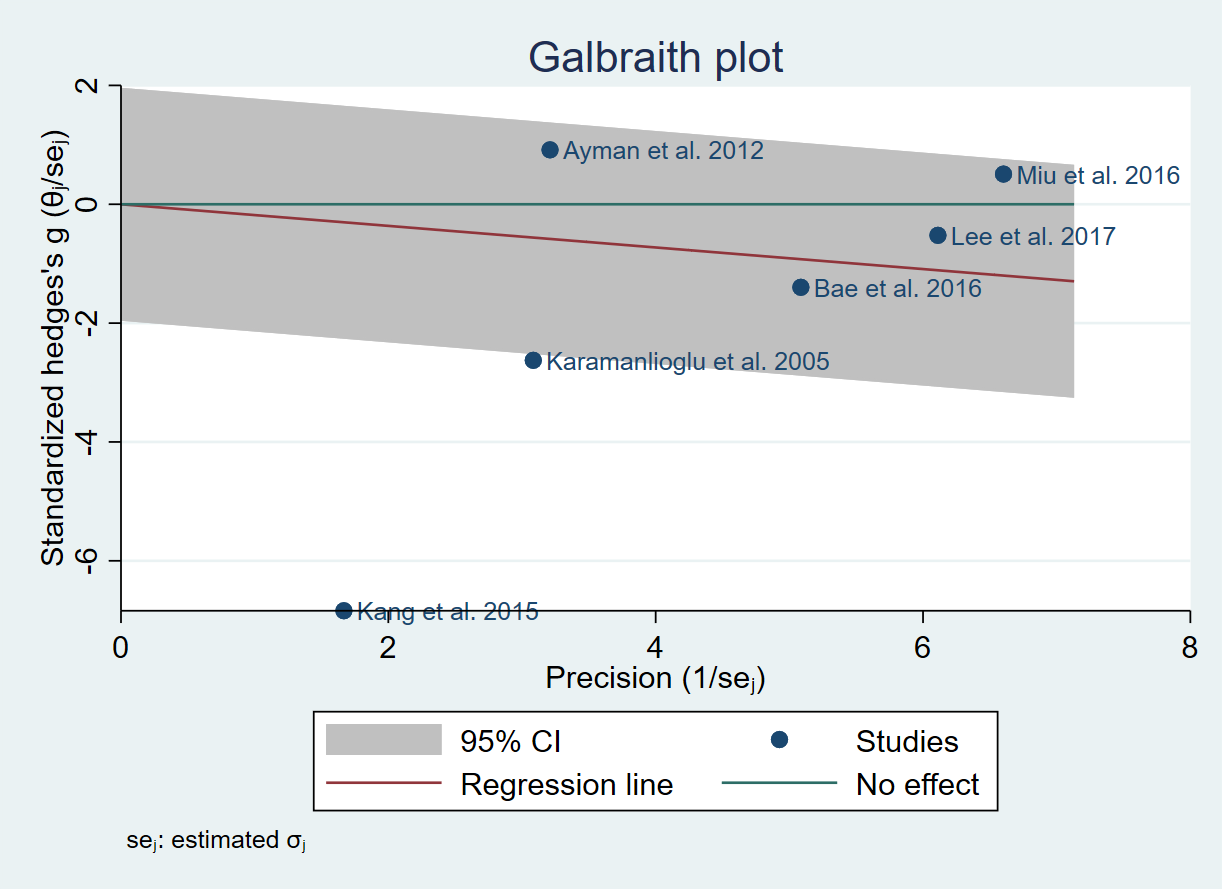


**Figure S7.** Galbraith plot of pain after 6-8 hours.


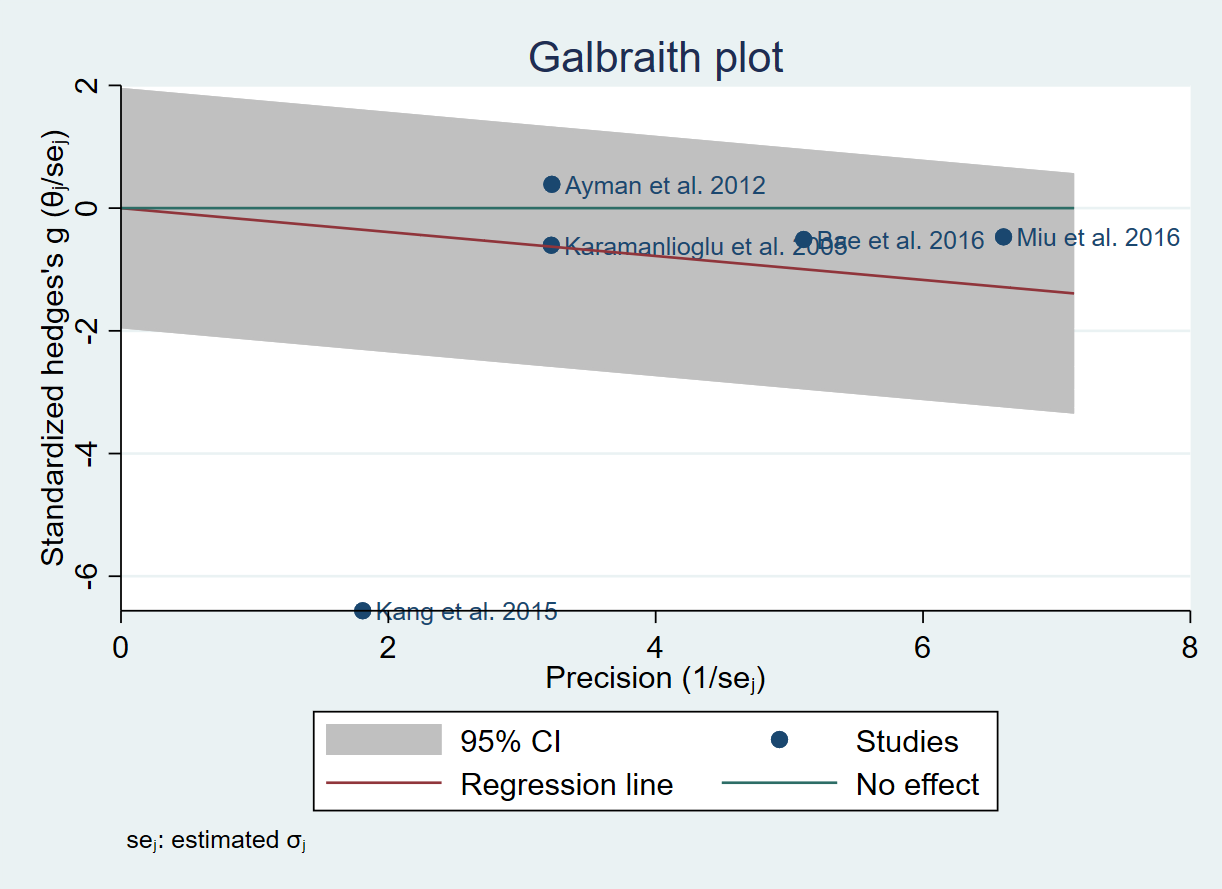


**Figure S8.** Galbraith plot of pain after 16-18 hours.


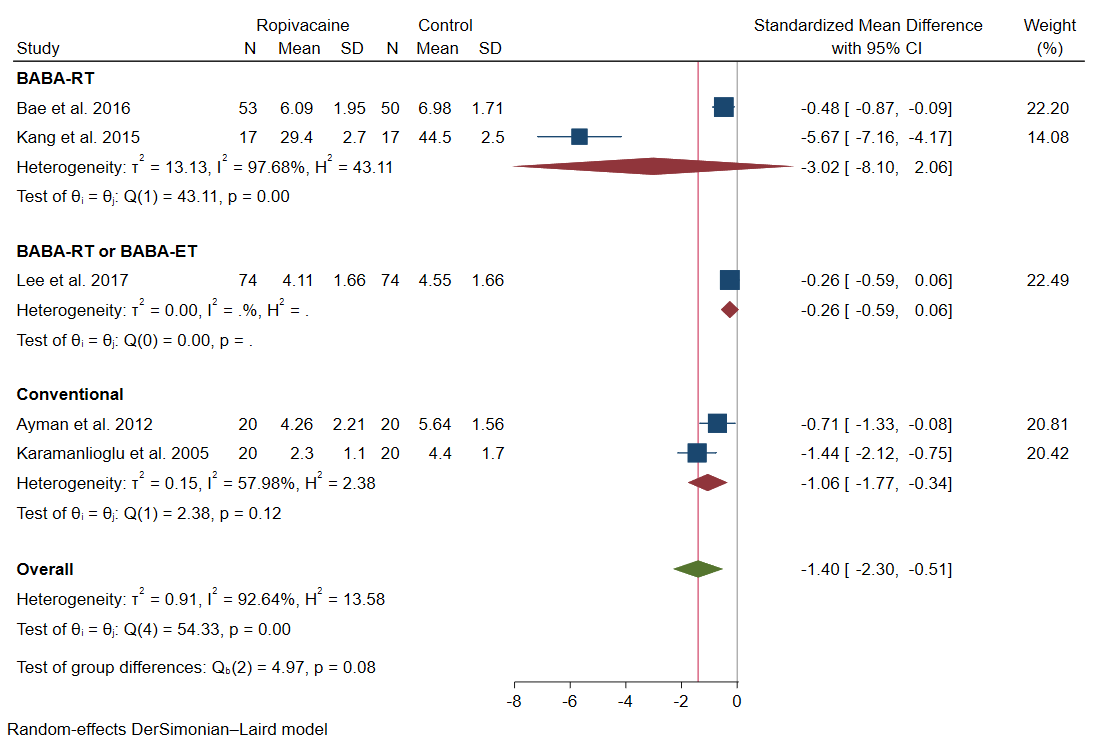


**Figure S9.** Subgroup analysis based on thyroidectomy type of pain after 1-2 hours.


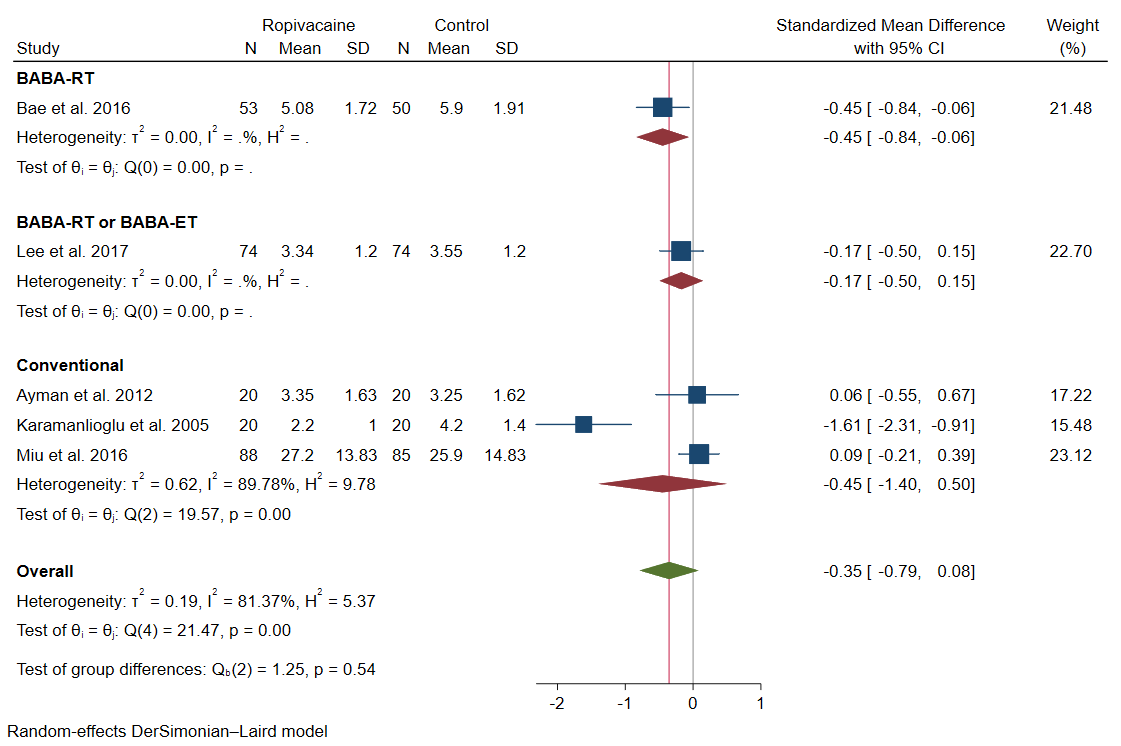


**Figure S10.** Subgroup analysis based on thyroidectomy type of pain after 4 hours.


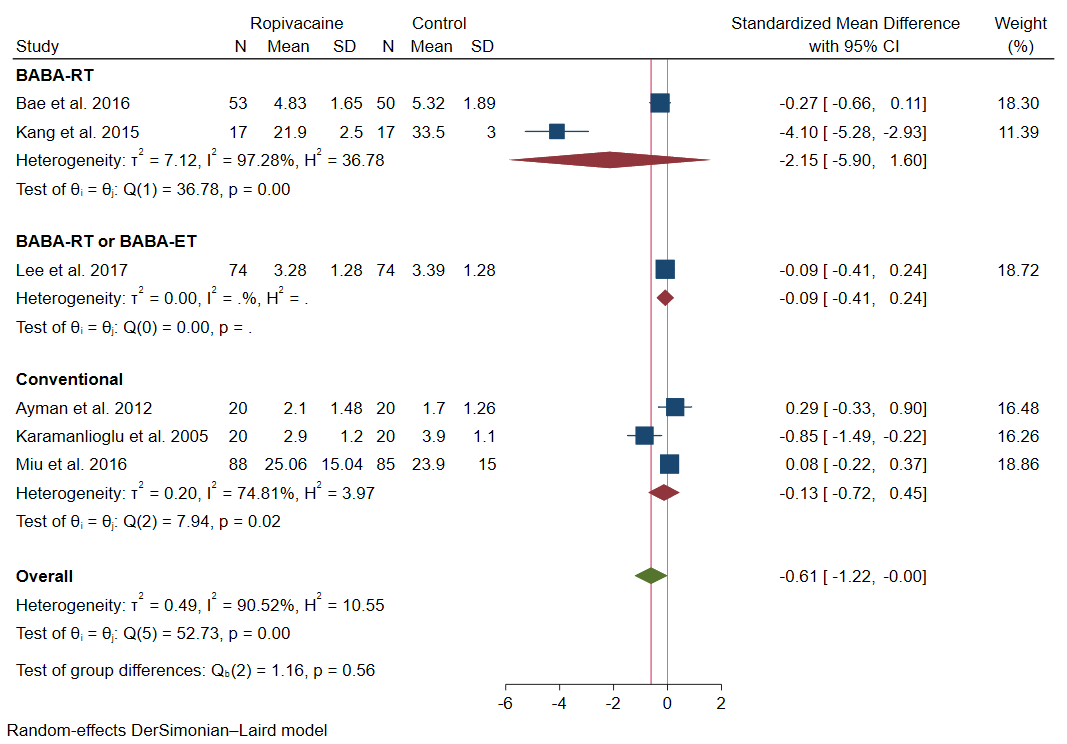


**Figure S11.** Subgroup analysis based on thyroidectomy type of pain after 6-8 hours.


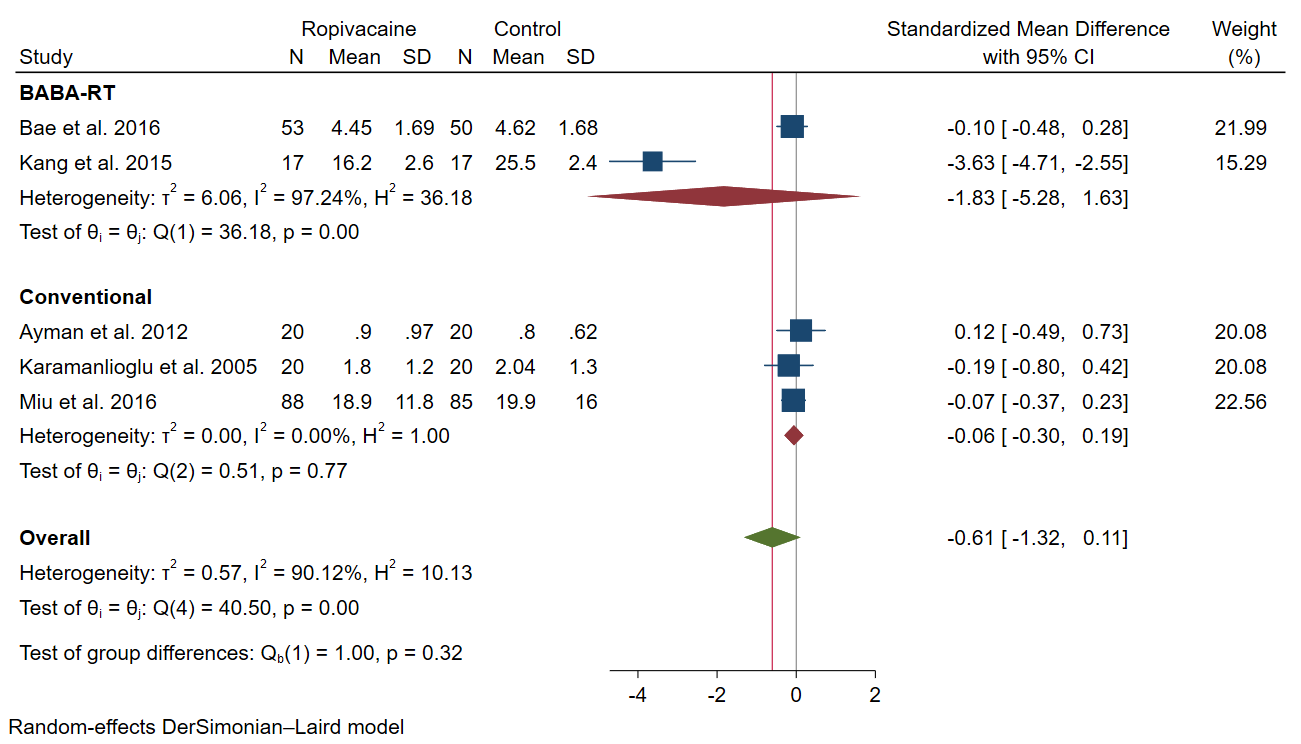


**Figure S12.** Subgroup analysis based on thyroidectomy type of pain after 16-18 hours.


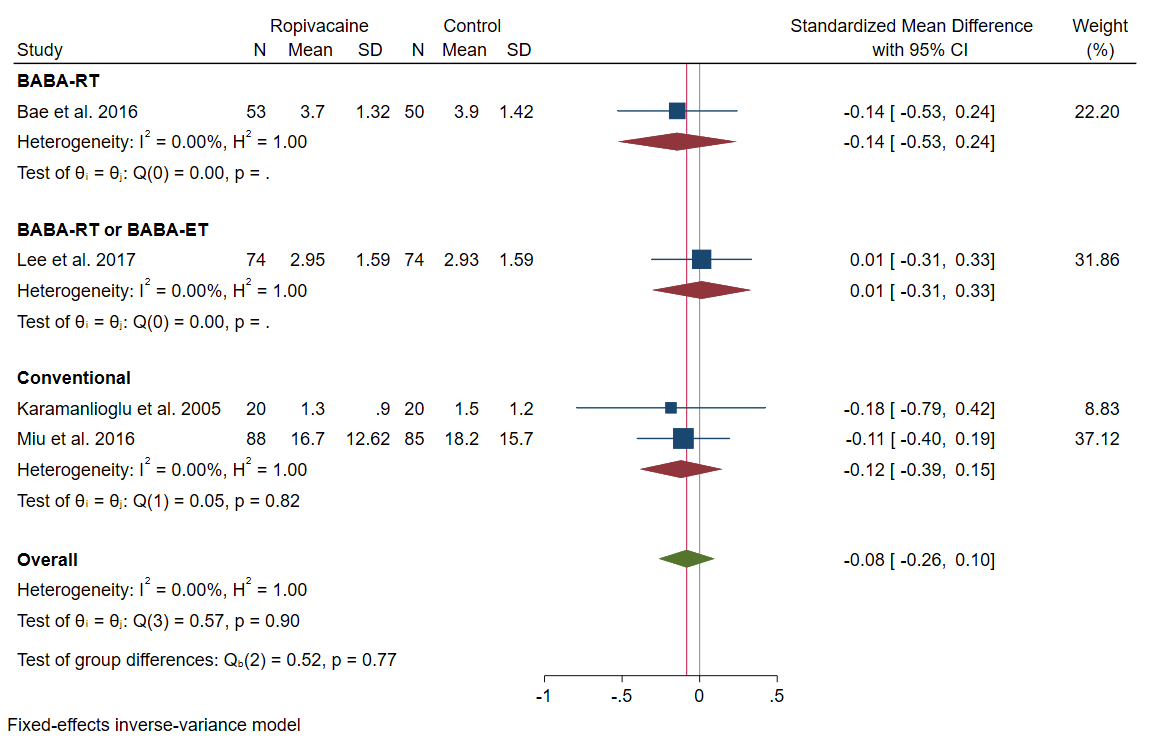


**Figure S13.** Subgroup analysis based on thyroidectomy type of pain after 16-18 hours.


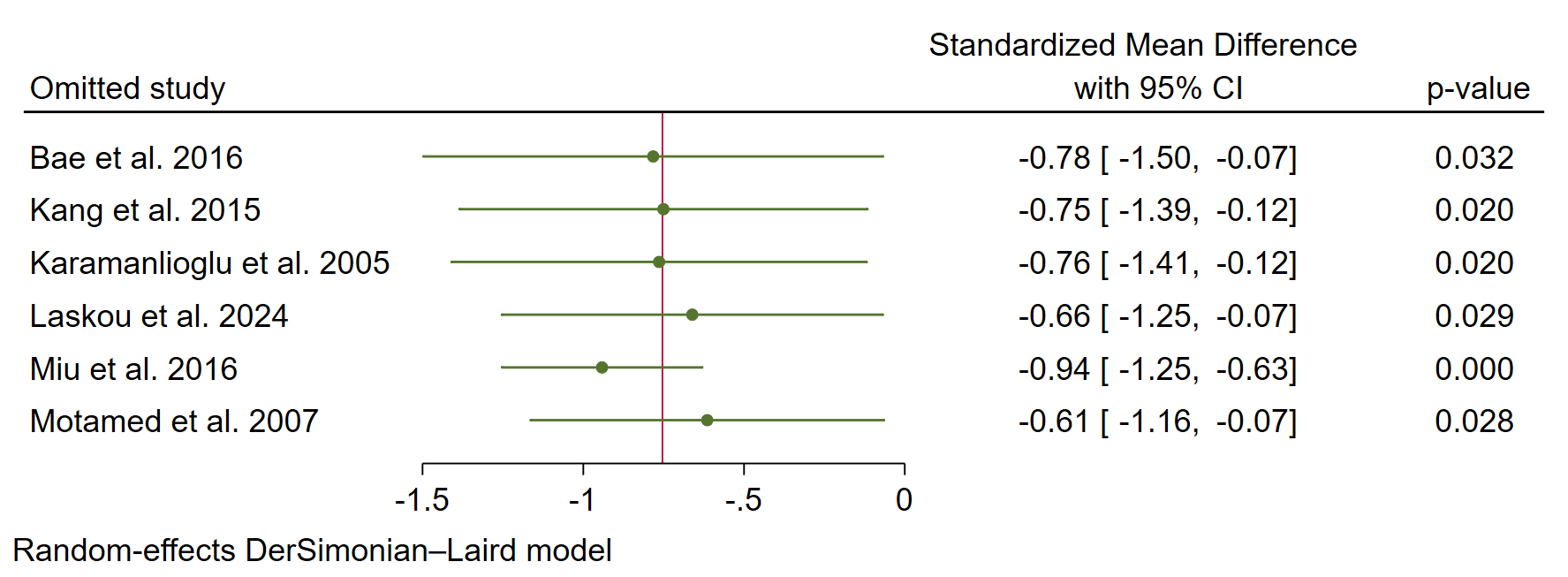


**Figure S14.** Leave-one-out sensitivity analysis of analgesia consumption.


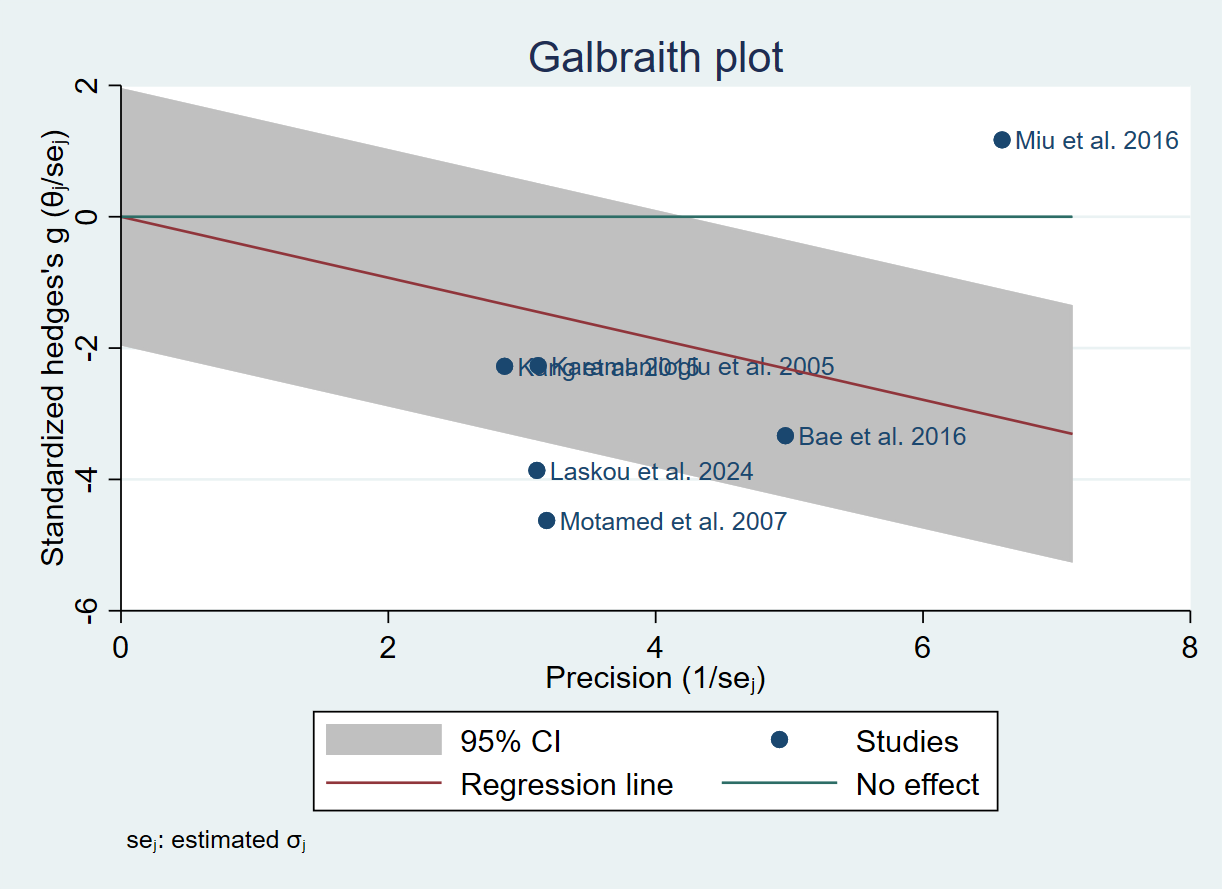


**Figure S15.** Galbraith plot of analgesia consumption.


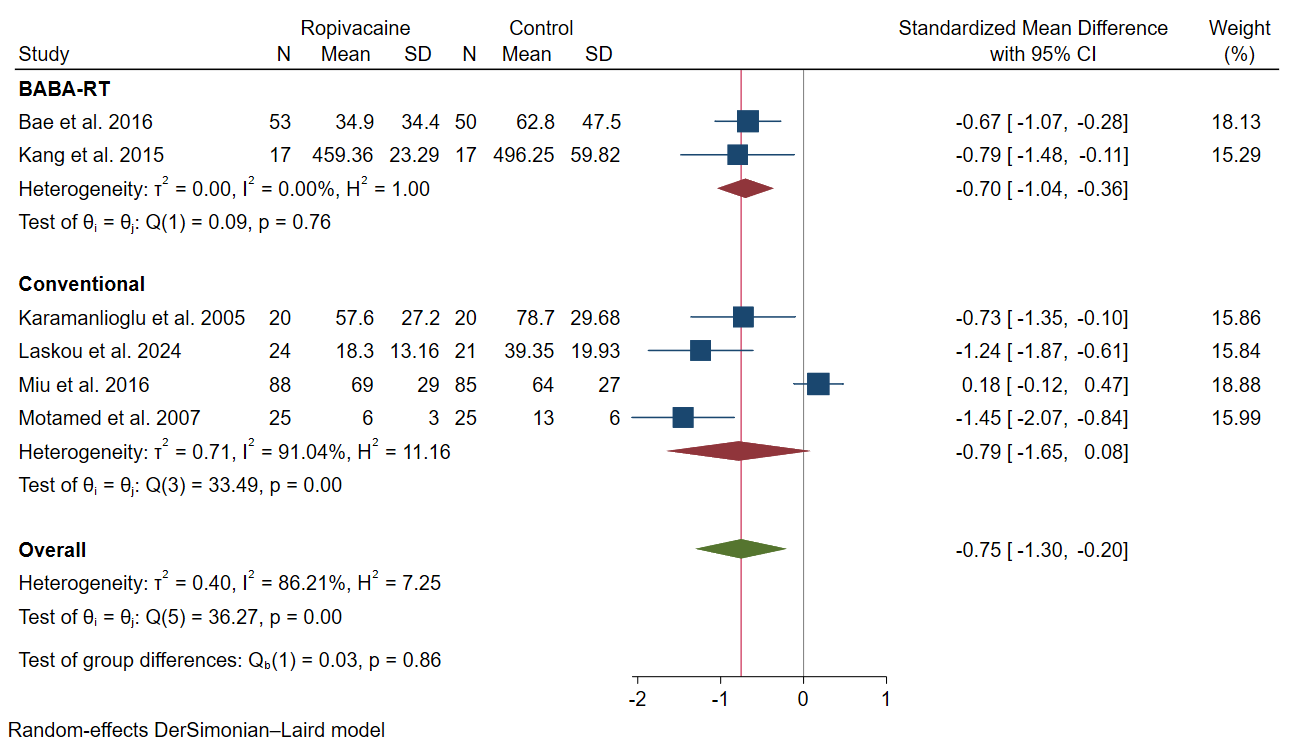


**Figure S16.** Subgroup analysis based on thyroidectomy type of analgesia consumption.


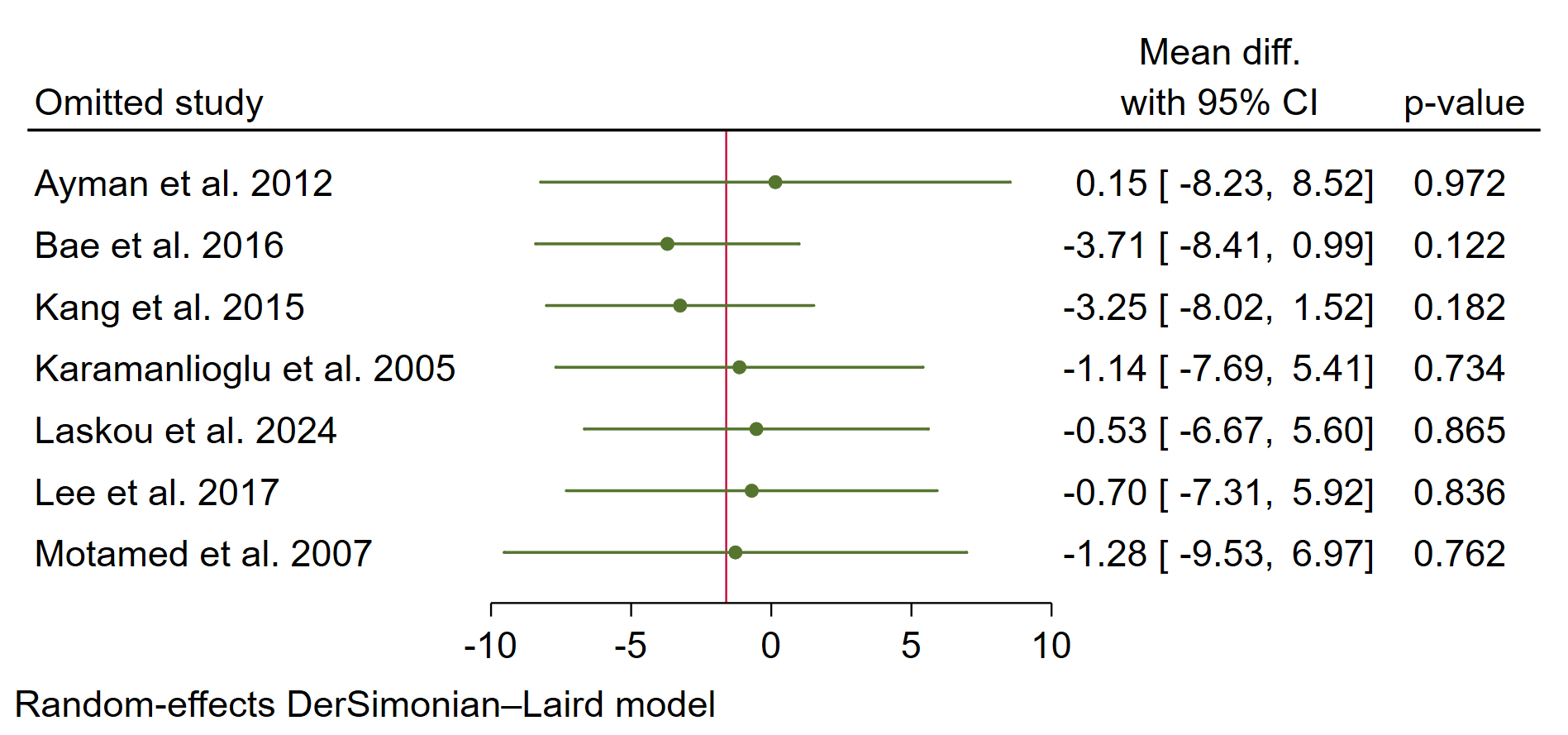


**Figure S17.** Leave-one-out sensitivity analysis of surgery duration.


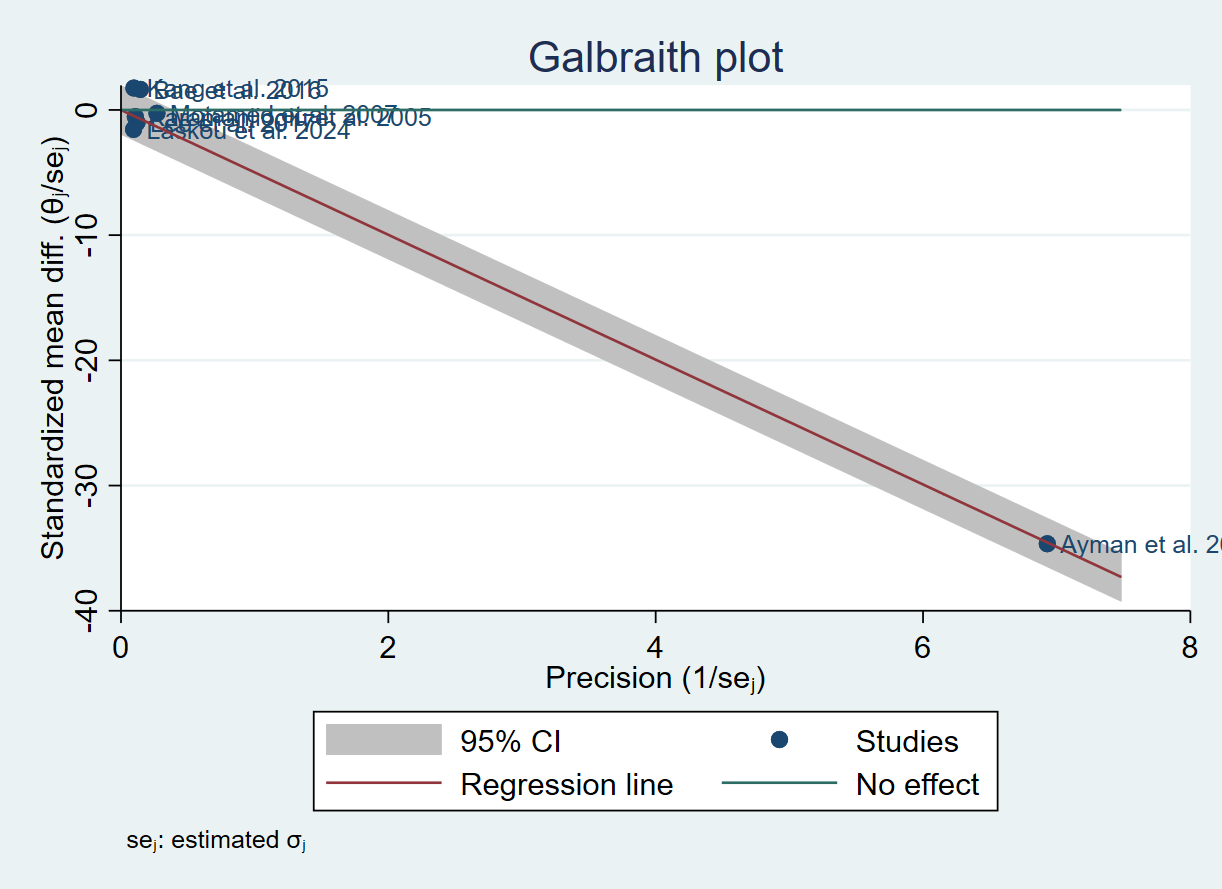


**Figure S18.** Galbraith plot of surgery duration.


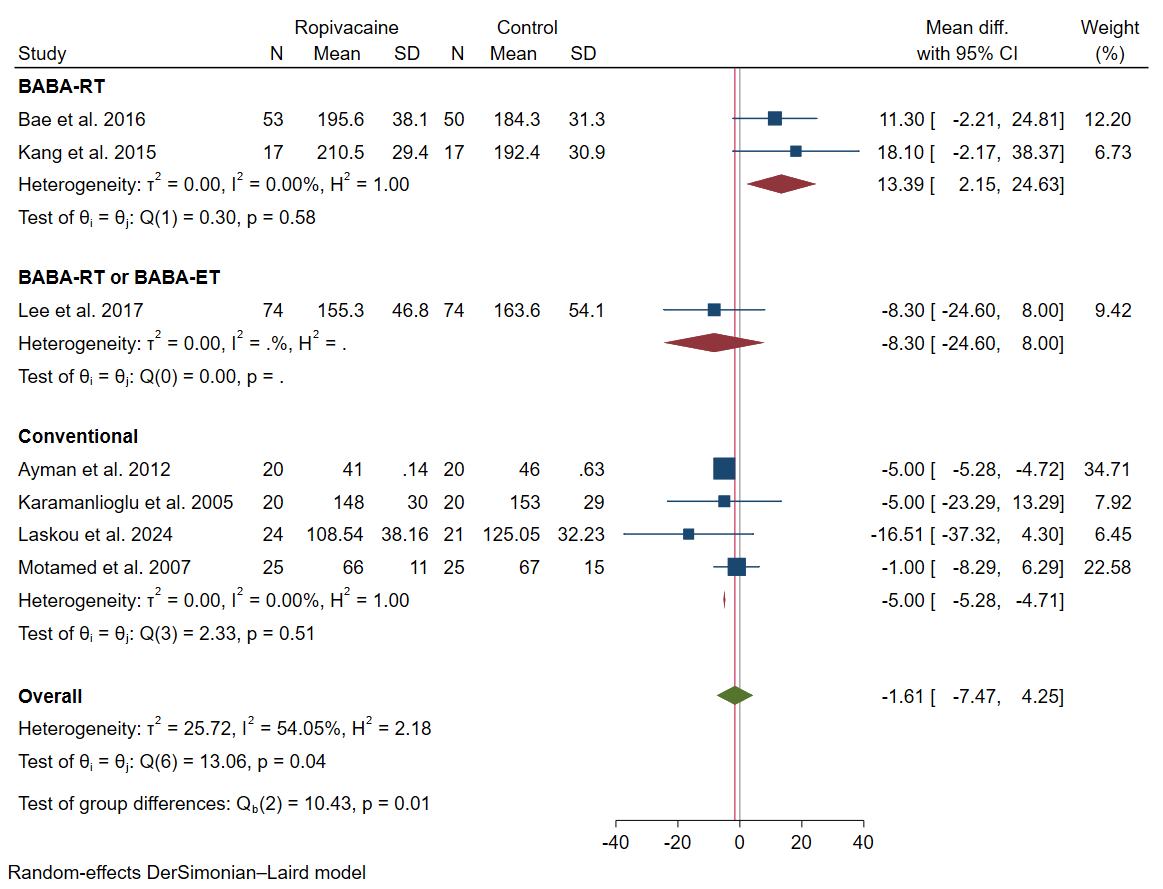


**Figure S19.** Subgroup analysis based on thyroidectomy type of surgery duration.


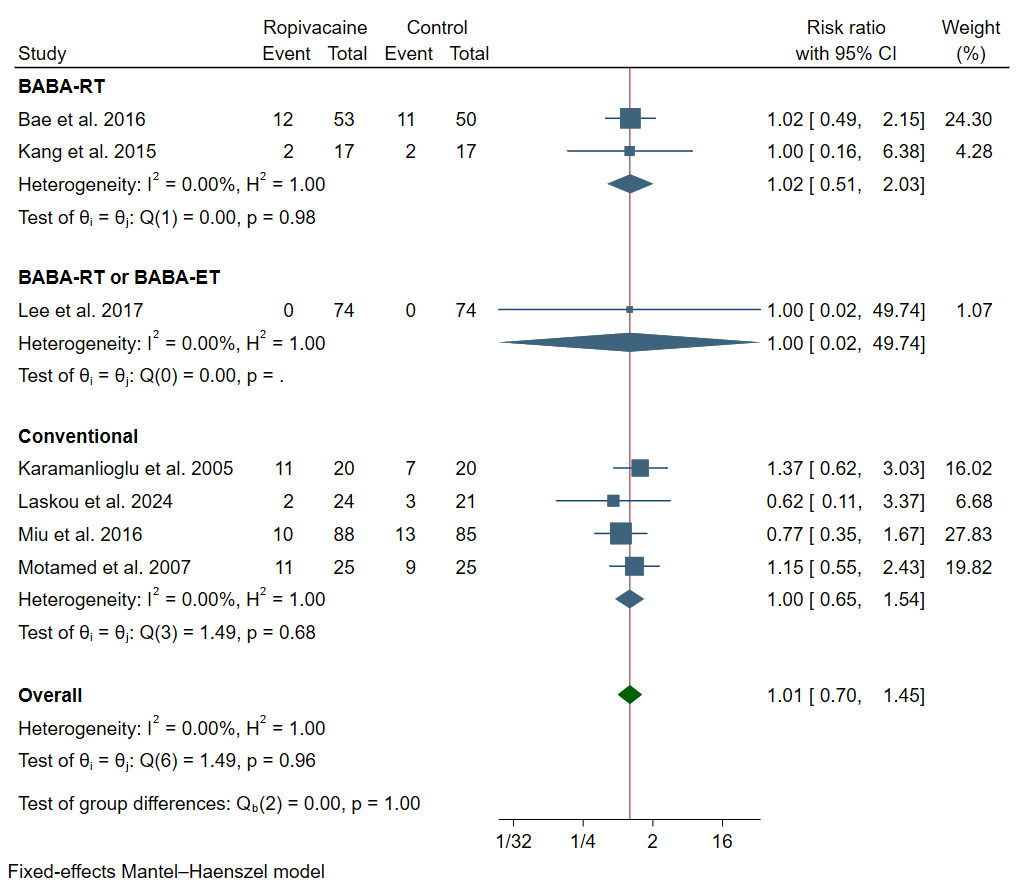


**Figure S20.** Subgroup analysis based on thyroidectomy type of PONV.
